# Nitric Oxide regulates mouth development in amphioxus

**DOI:** 10.1038/s41598-017-08157-w

**Published:** 2017-08-16

**Authors:** Giovanni Annona, Filomena Caccavale, Juan Pascual-Anaya, Shigeru Kuratani, Pasquale De Luca, Anna Palumbo, Salvatore D’Aniello

**Affiliations:** 1 Biology and Evolution of Marine Organisms, Stazione Zoologica Anton Dohrn di Napoli, Villa Comunale 1, 80121 Napoli, Italy; 2 Evolutionary Morphology Laboratory, RIKEN, Minatojima-minami 2-2-3, 650-0047 Kobe Hyogo, Japan; 3 RIMAR, Stazione Zoologica Anton Dohrn di Napoli, Villa Comunale 1, 80121 Napoli, Italy

## Abstract

The development of the mouth in animals has fascinated researchers for decades, and a recent study proposed the modern view of recurrent evolution of protostomy and deuterostomy. Here we expanded our knowledge about conserved traits of mouth formation in chordates, testing the hypothesis that nitric oxide (NO) is a potential regulator of this process. In the present work we show for the first time that NO is an essential cell signaling molecule for cephalochordate mouth formation, as previously shown for vertebrates, indicating its conserved ancestral role in chordates. The experimental decrease of NO during early amphioxus *Branchiostoma lanceolatum* development impaired the formation of the mouth and gill slits, demonstrating that it is a prerequisite in pharyngeal morphogenesis. Our results represent the first step in the understanding of NO physiology in non-vertebrate chordates, opening new evolutionary perspectives into the ancestral importance of NO homeostasis and acquisition of novel biological roles during evolution.

## Introduction

Nitric oxide (NO) is a small and highly diffusible signal molecule that is known to be involved in a wide range of important biological processes. Since its initial discovery as a modulator of vascular activities in mammals, NO has been found to participate in numerous physiological and developmental functions in a wide spectrum of organisms^1^. Our understanding of NO signaling has profoundly changed over recent decades. It was originally considered solely as a toxic substance, but nowadays, although harmful at high concentration, NO is believed to be an essential signaling molecule for living organisms. The function of this ambivalent gas depends on the precise balance between its production and consumption. When produced at high levels, for example during inflammation, NO may interact with cellular components, such as DNA, RNA, lipids, and proteins, leading to mutations and altered cell physiology that may lead to carcinogenesis^2–4^. On the other hand, NO deficiency can cause disorders of endocrine^5^, cardiovascular^6^, musculoskeletal^7^ and immune systems^8^.

The biosynthesis of NO is catalysed by the nitric oxide synthase enzymes (NOS), through two successive mono-oxygenation reactions, from L-Arginine to L-Citrulline with Nω-hydroxy-L-arginine (NOHLA) as an intermediate^9^. Mammalian genomes have three paralogous *Nos* genes with distinct expression patterns and specific functions^10, 11^: *NosI* or *neuronal Nos* (*nNos*); *NosII* or *macrophage inducible Nos* (*iNos*), and *NosIII* or *endothelial Nos* (*eNos*). All *Nos* genes share a very similar gene structure, with highly conserved intron number, position and phases. At the protein level, they only differ in the presence of the protein-interaction domain (PDZ) in NOSI, which is absent in both NOSII and NOSIII, and in the absence of the inhibitory loop in the region of FMN-binding domain exclusively in NOSII^12, 13^. Two of these genes, *NosI* and *NosIII*, are typically constitutively expressed, while *NosII* expression levels increase upon microbial infection, generating high and sustained amounts of NO^14^. Despite their given names indicating a tissue-specificity, all three *Nos* genes are, in fact, expressed in most tissues and organs. Therefore, we prefer to use the *NosI-II-III* nomenclature. In the central nervous system (CNS) the NO produced by NOSI is implicated in neurogenesis, synaptic plasticity, learning and memory^15^, while in the peripheral nervous system it is involved in the control of blood pressure, gut peristalsis and vasodilatation^14, 16^. NO derived from NOSII, primarily from macrophages, is essential for the control of inflammatory processes induced by intracellular bacteria or parasites^14^. Lastly, NO produced by NOSIII, which is the best characterized of the NOS proteins, is a homeostatic regulator of numerous essential cardiovascular functions, such as vasodilatation, inhibition of platelet aggregation and adhesion to the vascular wall, as well as inhibition of vascular inflammation^14^.

*Nos* genes are found in all living organisms, including bacteria^17^ and plants^18, 19^. During evolution, an ancestral proto-Nos gene was duplicated independently in several metazoan lineages, with a remarkable conservation in amino acid sequence and functional domains. Among chordates, the urochordate *Ciona intestinalis* possesses a single NosI-like gene containing a PDZ domain, and NO is a critical endogenous regulator of metamorphosis, apoptosis and ERK signaling^20–22^. As mentioned above, in tetrapod genomes, including mammals, three *Nos* paralogs have been identified^23–25^, while bony fish possess a variable *Nos* gene repertoire^23–25^.

Although the role of *Nos* genes is well established in urochordates and vertebrates (so-called olfactores), information available on cephalochordates (sister group of olfactores) is scattered in the literature. Presence of NOS was demonstrated prevalently in adult *Branchiostoma belcheri* tissues, mainly cerebral vesicle, muscle, endostyle and anus^26^, as well as nerve cord, wheel organ, epithelial cells of gut and midgut diverticulum, gill blood vessels, endostyle and ovary^27^. Later, NOS involvement in the immune system was demonstrated by Lin *et al*.^28^. The only attempt to study NOS during amphioxus development showed that the protein is present in the developing intestine (midgut and hindgut) and in the club-shaped gland of *Branchiostoma floridae* larvae^29^.

The foregoing studies were performed before the identification of the complete set of three *Nos* genes in the *B. floridae* genome: two NosI-like (*NosA* and *NosC*) and one NosII-like, so-called *NosB* ^23^. However, phylogenetic analyses showed that they are not one-to-one orthologs of the three vertebrate *Nos* genes, but they derived from an independent duplication in the cephalochordate lineage^23^. A comprehensive study aimed at discovering the different biological roles of all *Nos* genes during amphioxus embryogenesis was still missing.

In the present study, we have identified the *Nos* gene repertoire of three cephalochordate species and analysed their evolutionary history in comparison with other chordates. In addition, we have analysed the expression profiles of *Nos* genes in the European amphioxus, *Branchiostoma lanceolatum*. Lastly, we have investigated the nitric oxide localization as well as its biological functions during embryonic development in *B. lanceolatum*. We have found that NO is involved in the formation of the amphioxus mouth, acting in a sharp temporal window at early embryonic stages.

## Results

### *Nos* genes have independently duplicated in the lancelet lineage

**Figure 1:**
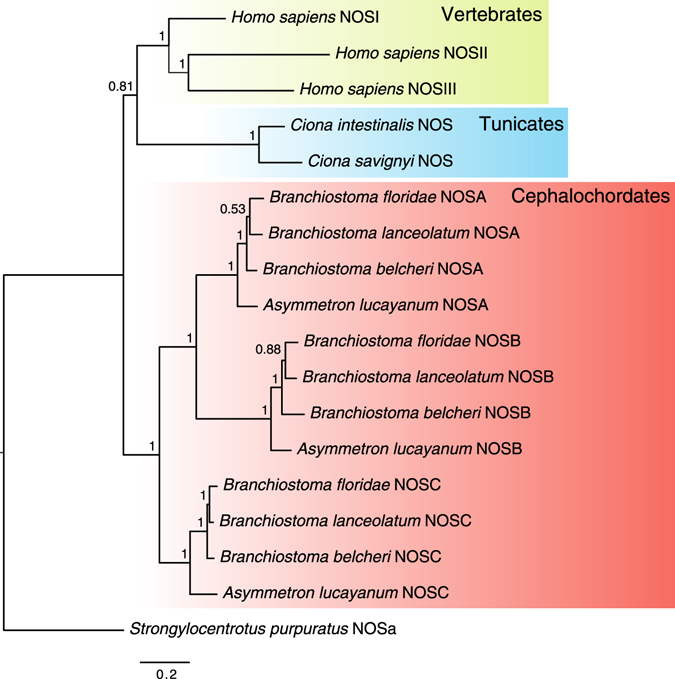
Phylogenetic analyses of NOS proteins in chordates. Bayesian inference-based phylogenetic tree of chordate NOS proteins. Numbers on nodes represent posterior probabilities values. The red box includes cephalochordate proteins, including 3 closely related species of *Branchiostoma* (*B. lanceolatum*, *B*. *floridae and B. belcheri*), plus *Asymmetron lucayanum*. The blue box contains two ascidian species, *Ciona intestinalis* and *Ciona savignyi*. Vertebrate NOS, represented here by *Homo sapiens*, are highlighted by the yellow box. The NOS protein of the sea urchin *Strongylocentrotus purpuratus* (Ambulacraria) was used as an outgroup.

Previous studies have highlighted the occurrence of several independent lineage-specific *Nos* gene duplications ﻿in metazoan evolution, including amphioxus *B. floridae* ^23^. In order to unravel whether the *Nos* expansion observed in *B. floridae* was present in other amphioxus species and to better define the *Nos* evolutionary history within the cephalochordates, we searched both genomic and transcriptomics databases for *Nos* genes in different cephalochordate species, from two different genera: *Branchiostoma* and *Asymmetron* (see Methods). We found three NOS paralogs in each of the analysed species: *B. belcheri*, *B. lanceolatum* and *Asymmetron lucayanum*. To confirm the orthologous relationships between cephalochordate *Nos* genes, we performed a phylogenetic analysis (Fig. 1). All *B. lanceolatum*, *B. belcheri* and *A. lucayanum* NOS proteins were closely related with high bootstrap values with the previously characterized *B. floridae* NOSA, NOSB and NOSC proteins, suggesting that the duplication events that resulted in the three cephalochordate *Nos* genes occurred in the last common ancestor of extant amphioxus (Fig. 1).

### Complementary *Nos* gene expression patterns during amphioxus development

**Figure 2:**
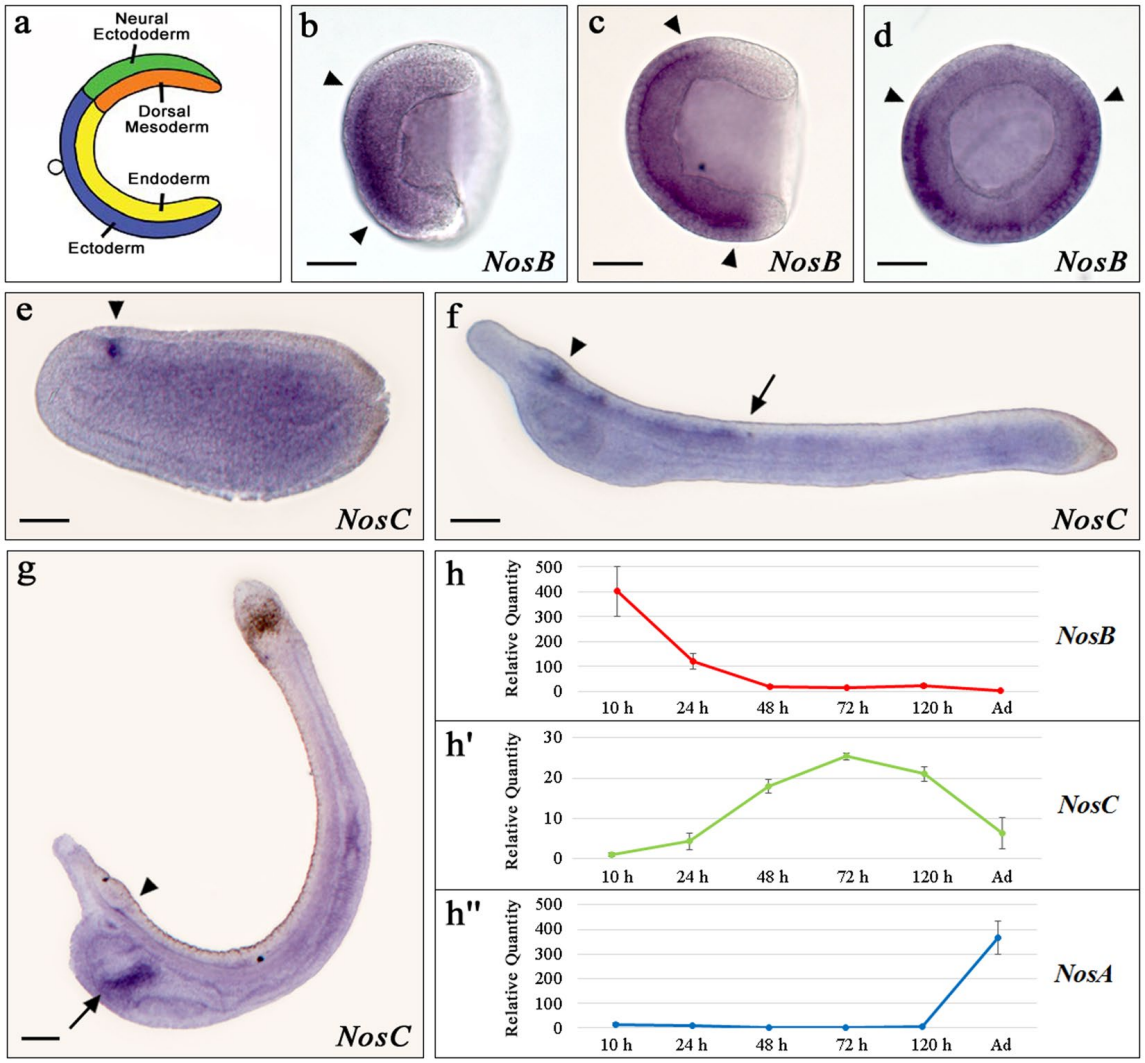
*Nos* genes expression patterns during *Branchiostoma lanceolatum* embryonic development. (**a**) Scheme of gastrula territory organization; (**b**) *NosB* expression at early gastrula stage, lateral view, (**c**) mid-gastrula stage, lateral view and (**d**) blastopore view [arrowheads indicate the limits of the positive signal]. (**e**) *NosC* gene expression at mid-neurula stage in neuropore (arrowhead); (**f**) at pre-mouth larva in brain vesicle (arrowhead) and in neural tube (posterior limit, arrow); (**g**) at larva 3 dpf in the brain vesicle (arrowhead) and club-shaped gland (arrow). In (**h** **-** **h**”) the ddPCR results of the three *Nos* genes in embryonic development are represented; P-value <0,05. Embryos orientation: anterior to the left (except 2d), dorsal to the top. Scale bars: 50 μm.

To examine whether *Nos* genes have a role in amphioxus development we characterized the temporal and spatial expression pattern of the three *B. lanceolatum Nos* genes. Droplet digital PCR (ddPCR) experiments showed a temporal complementary expression between the *B. lanceolatum NosB* and *NosC* genes (Fig. 2h-h’). During early developmental stages, strong *NosB* gene expression was detected. Initially *NosB* is expressed at gastrula stage [10 hours post fertilization (hpf)], followed by a decrease in expression levels at neurula stage (24 hpf) (Fig. 2h). At later stages of development *NosB* seems to completely switch off (Fig. 2h). *NosC* expression starts at pre-mouth larval stage (48 hpf) with the highest level of expression occurring at 3 days post-fertilization (dpf) larva (72 hpf) (Fig. 2h’). *NosC* expression levels decrease at 5 dpf larva (120 hpf) (Fig. 2h’). We were not able to detect discernible levels of *NosA* during the embryonic and larval stages analysed, but we observed expression in adult specimens (Fig. 2h”).

Expression patterns in whole-mount *in situ* hybridization preparations, were mostly in agreement with the trend observed in the ddPCR experiments: *NosB* was the first to be detected and its expression was limited to a few developmental stages. *NosB* expression was detected at early- (Fig. 2b) and mid-gastrula stage (Fig. 2c,d) in the endoderm, however it was absent in the area surrounding the blastopore, in the ectoderm and the dorsal mesoderm (Fig. 2b–d). We did not find any specific *NosB* signals at later developmental stages in whole-mount *in situ* hybridization experiments. Afterwards, following *NosB* down-regulation, we detected *NosC* expression from the mid-neurula stage onwards, which was specifically restricted to a few cells in the anterior part of the neural plate, slightly posterior to the neural pore (Fig. 2e). At the pre-mouth larval stage, *NosC* transcripts were detected in the anterior half of the neural tube, from the rostral part to the pigment spot (Fig. 2f). In 3 dpf larvae, the expression in the neural tube disappeared almost completely, remaining only in a few cells located in the most ventral and posterior part of the cerebral vesicle (Fig. 2g). At this stage, we also detected *NosC* expression in the club-shaped gland, which is closely connected to the pharyngeal area (Fig. 2g). The low levels of *NosA* expression revealed by ddPCR experiments were confirmed by the lack of any *in situ* hybridization signal in the developmental stages examined.

### Inhibition of the NO signaling prevents the formation of amphioxus mouth and gill slits

**Figure 3:**
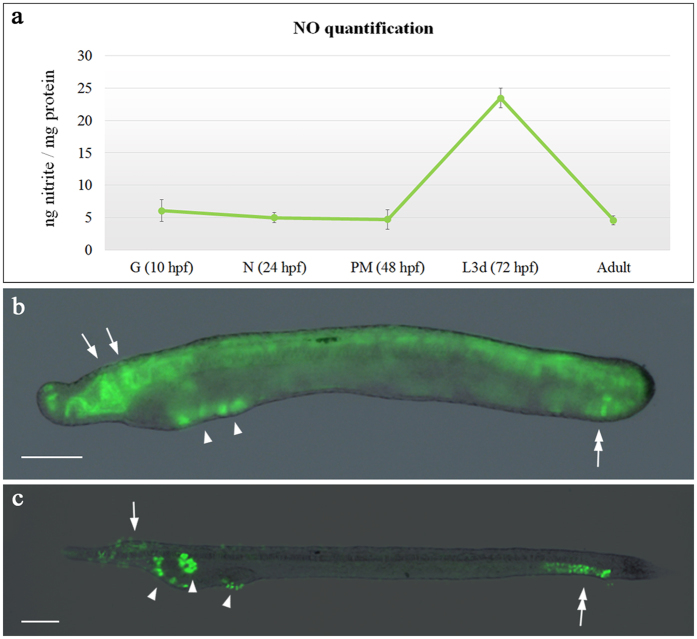
Nitrite quantification and nitric oxide detection in amphioxus embryos. The graph (**a**) shows the nitrite quantification during embryonic development and in adult obtained by Griess assay, the results are expressed as nmol of nitrite/mg of proteins. Nitric oxide localization by DAF-FM-DA at (**b**) 48 hpf and (**c**) 72 hpf. Arrows indicate nervous system; arrowheads show pharyngeal area, mouth and gill slits; tandem arrows indicate hindgut. Embryos orientation: anterior to the left, dorsal to the top. Scale bars: 50 μm.

Amphioxus *Nos* genes are expressed in different tissues during development. We assumed therefore that NO signaling could have an important role during embryogenesis of some, if not all, of those tissues. First, we measured endogenous NO levels during *B. lanceolatum* development by monitoring nitrite formation (Griess assay) to finely detect the exact localization of NO, independently of *Nos* transcript expression. From early to late development, we first observed a concentration of 6 nmol nitrite/mg protein at gastrula (10 hpf), followed by a decrease at neurula stage (24 hpf) and pre-mouth larvae (48 hpf), with 5 and 4.7 nmol nitrite/mg protein, respectively. At 72 hpf NO levels increased to 23.5 nmol nitrite/mg protein, decreasing again to average levels of 4.6 nmol nitrite/mg protein in adults (Fig. 3a). Next, using DAF-FM-DA, we detected where NO was localized in 48 and 72 hpf larvae. At 48 hpf larva, NO positive cells were abundant along all the neural tube (arrows in Fig. 3b) and in the most caudal extremity of the larvae, probably the future anal region (tandem arrows in Fig. 3b). Additionally, a strong NO fluorescent signal was observed in the corresponding area of the future mouth and gill slits (arrowhead in Fig. 3b). At 72 hpf, we observed a higher density of NO positive cells around the mouth, in the ventral part of the first gill slit and in the club-shaped gland (arrowheads in Fig. 3c). Later a punctate signal is still present in the rostral area as well as caudally in both the hindgut and anus (arrow and tandem arrows, respectively). In order to exclude the previously described endogenous GFP fluorescence in amphioxus^30, 31^, we checked green fluorescence emission in untreated *B. lanceolatum* larvae (negative control), showing a negligible non-specific signal at the same laser intensity as used for DAF-FM-DA experiments (Suppl. Fig. S1).

**Figure 4:**
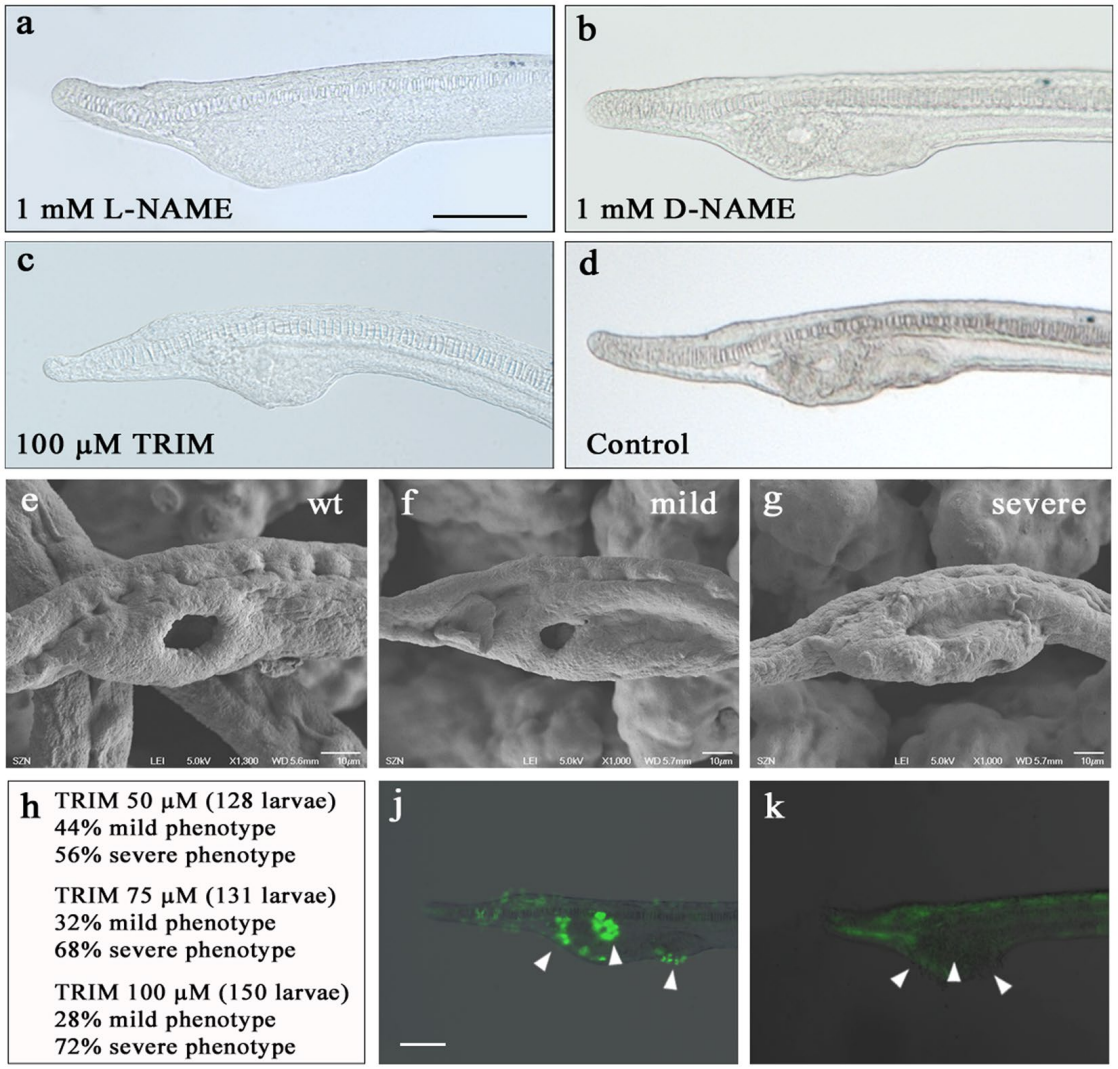
Drug treatments of amphioxus embryos. Amphioxus embryos were treated with L-NAME, D-NAME and TRIM, then the phenotype of 72 hpf larvae was observed. 1 mM L-NAME induces malformations in the mouth and gill slits area (**a**), not observed in the case of 1 mM of the D- enantiomer (D-NAME) (**b**). 100 µM TRIM-treated larvae show a phenotype similar to that observed with L-NAME (**c**). Control untreated larva kept in FSW (**d**). Next, 100 µM TRIM-treated larvae were observed by SEM. Control larvae in DMSO/FSW (**e**). TRIM-treated larvae with mild and severe phenotype (**f–g**). Percentages of larvae with mild and severe phenotypes at increasing drug concentrations, and the respective number of larvae observed (**h**). NO detection by DAF-FM-DA in untreated larvae mouth and gill slits are shown by arrowheads in (**j**). The absence of NO in mouth and gill slits of TRIM-treated larvae is indicated by arrowheads (**k**). Scale bars: 50 μm in (**a**–**d**) and (**k**–**j**).

We next investigated the role of NO during development, and thus the ontogenetic importance of NOS proteins. We experimentally reduced the endogenous NOS-produced NO with treatments using two different NOS inhibitors: Nω-Nitro-L-arginine methyl ester (L-NAME) and 1-(α,α,α-trifluoro-o-tolyl)-Imidazole (TRIM) at different temporal windows^22, 32, 33^ (see Methods; Suppl. Fig. S2 and Fig. 4). Treatments with a concentration of 100 μM of L-NAME resulted in normal larvae in each of the experimental times assayed, indistinguishable from wild type control treatments (Fig. 4d). Experiments with 1 mM L-NAME added at neurula stage (24 hpf) and maintained to 3 dpf larva stage resulted in larvae in which the mouth and gill slits did not form, without affecting the other morphological features (Fig. 4a). In the experiments performed in other temporal windows, the L-NAME treatment did not induce any body malformations, except when present throughout development from gastrula to larva, giving rise to an abnormal body plan (Suppl. Fig. S2). Increasing the L-NAME concentration to 10 mM produced larvae with an abnormal body plan, presumably due to the toxicity of high drug concentrations rather than a specific effect. As a control for L-NAME treatments we used D-NAME, the inactive D- form enantiomer, at the same experimental conditions. D-NAME did not affect amphioxus development (Fig. 4b).

To understand whether the observed alteration of the buccal area was due to the toxicity of the L-NAME or to a specific inhibition effect on NOS activity, we performed a second independent series of *in vivo* treatments using a different NOS inhibitor (TRIM). Treatments with 50, 75 and 100 µM TRIM from neurula stage until day 3 of development causes alterations mainly in the mouth and gill slits area of the amphioxus larvae (Fig. 4c, compare with Fig. 4a). There was a significant increase in the proportion of larvae with severe phenotype (no mouth opening) in a dose dependent manner (50 to 100 μM) (Fig. 4h). To better characterize the head malformation, we treated larvae with 100 μM TRIM and then examined by scanning electron microscopy (SEM). While the general animal morphology was unaffected, mouth and gill slits structures were malformed. We classified this phenotype according to its severity, as either mild (reduction of the mouth opening, Fig. 4f) or severe (absence of the mouth opening, Fig. 4g). We further investigated if these morphological alterations positively correlated with a decreased intracellular NOS-produced NO. NO localization detection by DAF-FM-DA in TRIM treated embryos showed that the mouth absence was associated with the disappearance of NO fluorescent signal (arrowheads in Fig. 4k), which in contrast is present in the untreated animals (arrowheads in Fig. 4j).

## Discussion

NO has probably played a crucial role in the early history of life on Earth providing protection to primitive microorganisms, neutralizing the aggressive oxidative effect of rising ozone levels in the atmosphere. NO does not require carrier molecules to cross cell membranes, and can easily reach intracellular targets by diffusion even over large body distances. During animal evolution, NO has acquired several novel functions beyond the mere enhancement of survival^34^. With this in mind, we tried to gain insight into the evolutionary history of *Nos* genes in chordates, particularly studying in detail both the *Nos* genes repertoire and putative functions of NO in the cephalochordate amphioxus *B. lanceolatum*. Although we previously studied amphioxus *Nos* gene relationships with other metazoan *Nos* genes^23^, their evolution within the cephalochordate clade was still unclear. We have confirmed the presence of three *Nos* genes, *NosA*, -*B* and -*C*, in other *Branchiostoma* species, *B. lanceolatum* and *B. belcheri*, as well as in an *Asymmetron* species, *A. lucayanum. Branchiostoma* and *Asymmetron* represent lineages diverged directly from the last common ancestor of extant cephalochordates^35^, and therefore comparisons between them are informative to determine the condition of the latter (Fig. 1). Our phylogenetic analysis clearly shows that both *Branchiostoma* and *Asymmetron Nos* paralogs are one-to-one orthologous *Nos* genes, suggesting that the duplication events that originated cephalochordate *NosA*, *-B* and *-C* paralogous genes, happened in their last common ancestor (Fig. 1).

While we did not detect *NosA* expression, we found a complementary expression of *NosB* and *NosC* (Fig. 2h-h’). *NosB* was highly expressed during gastrulation (Fig. 2b–d). Interestingly, NO is thought to be involved in cell division and cell motility during gastrulation in *Drosophila* and *Xenopus* ^36, 37^. Therefore, it is tempting to hypothesize that also in amphioxus NOSB may exert important roles during gastrulation. NO levels in whole embryos were in general concordant with *Nos* expression levels, suggesting that NOS likely exert their roles by means of NO production and, importantly, in a regulated fashion during amphioxus development. *NosC* expression starts at the neurula stage in a few cells in the most anterior part of the neural plate (Fig. 2e), then expands from this most anterior region to the pigmented spot at pre-mouth larvae (Fig. 2f) and later gets restricted to a few cells of the cerebral vesicle and to the club-shaped gland at 3 dpf larvae (Fig. 2g). Although direct comparison between *Nos* expression patterns in nervous systems between vertebrates and cephalochordates is difficult^38^, it seems that a similarity exists between zebrafish *NosI* gene (expressed at 24 hpf in differentiating neurons and then in the major areas of the brain) and amphioxus *NosC* gene expression^39, 40^. Moreover, our NO localization experiments showed that NO partially coincides with the expression patterns of *NosC* gene at pre-mouth larvae, like in the cerebral vesicle and neural tube. Altogether, these results suggest a putative involvement of *NosC* in amphioxus CNS function, although further experiments are needed to find out what this function might be.

Besides the expression in the CNS, we observed a peak of *NosC* transcript levels (Fig. 2h’) that was mainly localized, together with significant amounts of NO, in the pharyngeal area in 3-dpf larvae (compare Figs 2g and 3c). This stage represents a pre-metamorphic developmental phase possessing an already formed neural tube, functional muscles and an open mouth on the left side of the body, in addition to other embryonic transitory organs: endostyle, pre-oral pit and club-shape gland. Interestingly, the presence of an intensively innervated portion of the pharynx in pre- and post- metamorphic larvae has been demonstrated, indicating that the club-shaped gland, the pre-oral pit and the endostyle are probably involved in important morphological processes in amphioxus mouth opening and rostral metamorphosis^41^. Here we showed *NosC* expression in the club-shaped gland, therefore we assume a possible involvement of this organ in the morphogenesis of pharyngeal structures.

NO is the final product of the Kinin-Kallikrein pathway, which in adult vertebrates usually participates in inflammation processes, as well as in the regulation of blood pressure. Recently, this pathway has been proposed to be active in the so-called “extreme anterior domain” of *Xenopus* and zebrafish embryos and to be essential for craniofacial development^42^. *NosI*-morphants and TRIM treated frog embryos at neurula stage developed abnormal cranio-facial structures with a complete absence of the mouth^42^. Addition of a NO donor led to a complete rescue of the facial development, demonstrating that NO is necessary for mouth development in vertebrates^42^. Decreasing endogenous NO levels in amphioxus upon NOS inhibition, similarly to vertebrates, has led to the development of amphioxus larvae with a compromised pharyngeal structure, showing severely reduced or absent mouth and gill slits (Fig. 4). Interestingly, this developmental abnormality was observed only when the NO depletion was carried out during a sharp temporal window. The capability of the embryo to recover the correct morphology after the removal of the drug at 36 hpf (that is after 12 hours of treatment) allowed us to demonstrate the precise time interval in which NO is likely to have a role in mouth and pharynx formation: between 36 and 48 hpf. This suggests that also in amphioxus the embryonic origin of the prospective chordate primary mouth is under direct NO control during the neurula stage. Because of its characteristics, the amphioxus mouth still represents a longstanding enigma with regards to its evolutionary origin, homology relationships and differences with other chordate mouths. Recently, Nodal signaling in amphioxus has been shown to control left-right asymmetric development, in which the mouth is a prominent feature^43^. An independent study proposed that a mesodermal vesicle becomes intimately juxtaposed to the nascent mouth at the early larval stage under the control of several genes belonging to the Nodal-Pitx signaling pathway^44^.

**Figure 5:**
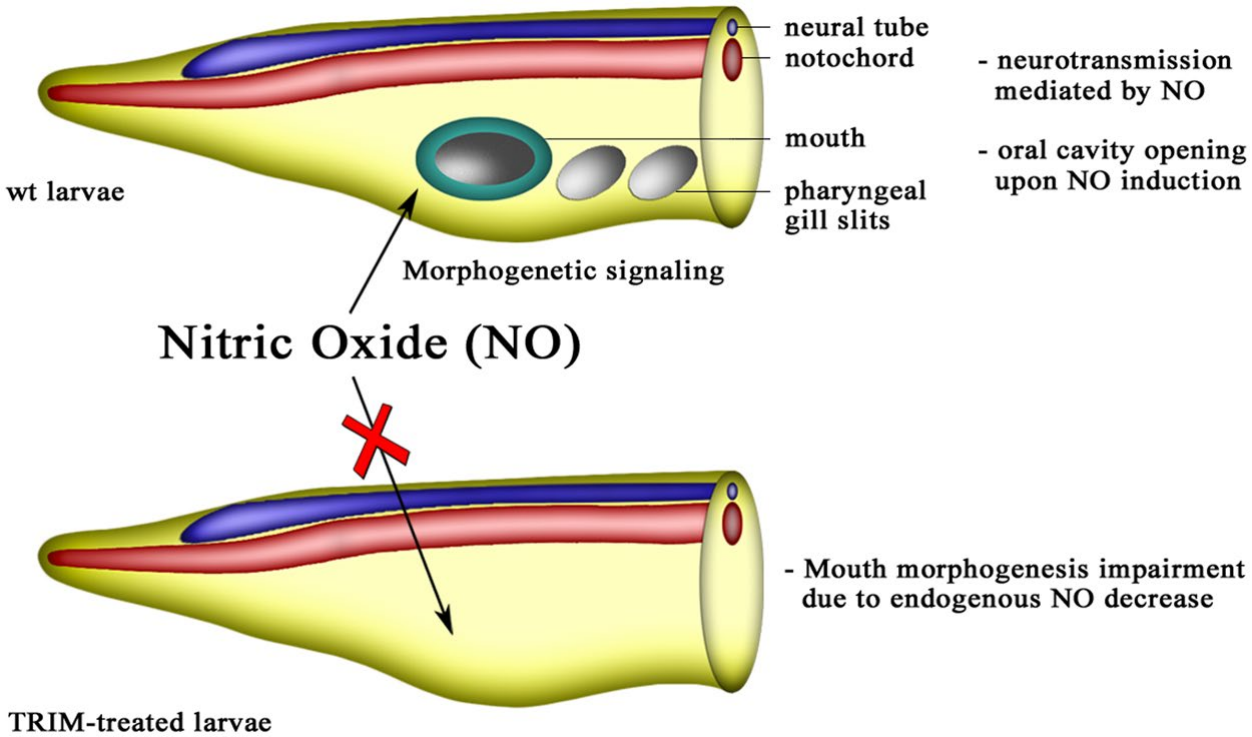
Nitric Oxide role during amphioxus larval development. Schematic representation of the rostral part of amphioxus larvae indicates possible involvement of NO in mouth development. The conspicuous depletion of endogenous NO by NOS inhibition (TRIM-treated larvae) leads to an abnormal phenotype without a mouth.

In conclusion, the results of the present study showed for the first time the crucial role of NO as an endogenous regulator of mouth formation in amphioxus (Fig. 5). The balance of NO levels in the pharynx-surrounding area is likely to be a prerequisite for the correct morphogenesis of the mouth. Future studies are needed to investigate if there is any relationship between NO and Nodal-Pitx pathway in amphioxus mouth morphogenesis, and to clarify whether the Kinin-Kallikrein signaling, discovered in vertebrates, is conserved in amphioxus.

## Methods

### Ethics Statement

Adult amphioxus specimens (*B. lanceolatum*) were collected from an endemic population of the Gulf of Naples (Italy), according to the authorization of Marina Mercantile (D. Lgs. 09/01/2012, n.4). All procedures were in compliance with current available regulations for the experimental use of live animals in Italy.

### Animal care and embryo collection

Animals were kept in an open circulating system reproducing natural thermal and light conditions, development of the gonads was periodically monitored. Ripe males and females were induced to spawn and the embryos were cultured at 18 °C as described in literature^45^. Embryos used for total RNA extraction were collected and fixed in EUROzol (Euroclone) and stored at −80 °C until used. For *in situ* hybridization experiments embryos were transferred into 4% paraformaldehyde (PFA) in MOPS/EGTA solution (0.1 M MOPS pH 7.5; 2 mM MgSO_4_; 1 mM EGTA; 0.5 M NaCl in DEPC-H_2_O) and dehydrated in ice-cold 70% EtOH in DEPC water and kept at −20 °C until used.

### Identification of lancelet *Nos* genes and phylogenetic analysis

*B. lanceolatum Nos* genes were annotated in the genome draft version Bl71nemr, kindly provided by the “*Branchiostoma lanceolatum* genome consortium”. *B. lanceolatum Nos* gene sequences are available in the Suppl. Fig. S3. *B. belcheri Nos* genes were identified from the automated predictions of NCBI (corresponding accession numbers in Suppl. Table S4). To find *A. lucayanum Nos* genes we screened a previously published transcriptome assembly^46, 47^ (DDBJ/EMBL/NCBI accession numbers GESY00000000 and GETC00000000). Accession numbers of *A. lucayanum* transcripts corresponding to each *Nos* paralog are available in Suppl. Table S4. Briefly, we performed TBLASTN searches using amino acid sequences of the three NOSA, NOSB and NOSC proteins from *B. floridae*, and candidate scaffolds, contigs or transcripts were further analysed by means of GeneWise2 as implemented in the EBI website^48, 49^ and manual curation. Other NOS proteins included in the phylogenetic analysis were collected from public databases such as Ensembl and NCBI (see Suppl. Table S4 for accession numbers).

For the phylogenetic analysis, NOS amino acid sequences were aligned using the MUSCLE algorithm^50^ as implemented in MEGA v7, release 7161111-i386^51^ with default parameters, and saved in FASTA format. The alignment was trimmed by trimAl version 1.2rev59^52^, using the ‘-automated1’ parameter. The trimmed alignment was then formatted into a nexus file using readAl^50^ (bundled with the trimAl package) (Suppl. File S5). A Bayesian inference tree was inferred using MrBayes 3.2.6^53^, under the assumption of an LG + I + G evolutionary model. Two independent MrBayes runs of 1,000,000 generations, with 4 chains each, were performed. The tree was considered to have reached convergence when the standard deviation was stabilized under a value of <0.01. A burn-in of the 25% of the trees was performed to generate the consensus tree (750,000 post-burn trees).

### Cloning and riboprobes preparation

Total RNA, from *B. lanceolatum* adult tissues (for *NosA)* or embryos (for *NosB* and *NosC)*, was extracted using EUROzol (EuroClone) reagent and chloroform, and precipitated from the aqueous phase with isopropyl alcohol. cDNA was synthesized from 0.5–1 µg of total RNA using the SMART PCR cDNA Synthesis Kit (Clontech). Different fragments corresponding to the three *B. lanceolatum Nos* genes were amplified using specific primers, designed in order to avoid cross-hybridization among the three paralogous genes (Suppl. Table S6), and cloned into the pGEM-T Easy Vector (Promega). Antisense Digoxygenin-UTP riboprobes were synthesized using the SP6 or T7 RNA polymerases and the (DIG) RNA Labeling Kit (Roche).

### Droplet digital polymerase chain reaction (ddPCR)

Expression profiles of *B. lanceolatum Nos* genes were analysed by Droplet digital PCR (ddPCR) in biological triplicates. Total RNA was extracted from embryos at different developmental stages: gastrula (10 hpf), middle neurula (24 hpf), pre-mouth larva (48 hpf), 3 dpf and 5 dpf larvae. Approximately 500 ng of total RNA extracted from each time point was reverse transcribed to cDNA using Super Script Vilo kit (Invitrogen). cDNA (approx. 3 ng) was mixed with 10 µl of 2X ddPCR Evagreen Supermix, 0.5 pM of each primer and nuclease-free water to a total reaction volume of 20 µl, then loaded into a sample well of a DG8 Cartridge for the QX200/QX100 droplet generator, according to the QX200/QX100 Droplet Generator Instruction Manual. Thermal cycling was then performed on the droplets using the C1000 Touch Thermal Cycler with 96-deep well reaction module according to the following protocol: enzyme activation at 95 °C for 10 min (1 cycle), denaturation at 94 °C for 30 sec followed by annealing/extension at 60 °C for 30 sec (40 cycles), enzyme deactivation at 98 °C for 10 min (1 cycle) followed by hold at 4 °C. All reagents and equipment used for ddPCR were from Bio-Rad Laboratories. The absolute gene expression level per well for the probes and reference genes were quantified using QuantaSoft software. The gene expression values for each sample were normalized to the housekeeping gene *Ribosomal protein L32* (*RPL32*)^54^ and reported as relative quantity compared to the lowest expression level of each *Nos* gene, respectively. The results for the three *Nos* genes at each developmental stage were subjected to Student t-test; a *P*-value of less than 0.05 was considered significant.

### Whole-mount *in situ* hybridization

For whole-mount *in situ* hybridization, embryos were re-hydrated in 1X PBT, treated with proteinase K (5 µg/ml) to facilitate riboprobe penetration; the reaction was stopped by adding 4 μl of 10% glycine and then washed with 2 mg/ml glycine in a phosphate buffered saline solution containing 0.1% Tween-20 (PBT). The embryos were re-fixed in PBT containing 4% PFA for 1 h at RT, subsequently washed in 0.1 M triethanolamine and then with 0.1 M triethanolamine plus acetic anhydride, to prevent non-specific background staining. Embryos were washed with PBT several times, pre-hybridized at 60 °C for 1 h and finally hybridized by shaking at 65 °C overnight, in DEPC-H_2_O hybridization buffer (50% deionized formamide; 100 μg/ml Heparin; 5X SSC; 0.1% Tween-20; 5 mM EDTA; Denhardt’s 1 mg/ml; yeast RNA 1 mg/ml). The day after post-hybridization, washes were performed in decreasing concentrations from 5X to 2X of SSC 50% formamide/dH_2_O at hybridization temperature and then at room temperature in decreasing concentrations of SSC, from 2X to 0.2X in dH_2_O. An RNAse step at 37 °C was included. Embryos were incubated overnight in primary antibody (anti-DIG AP, Roche), pre-adsorbed at 1:3000, with rocking at 4 °C. The signal was revealed at room temperature using BM-Purple substrate (Roche). Afterwards embryos were washed several times in PBT, postfixed in 4% PFA for 20 min, mounted in 80% glycerol in PBS, and photographed under Axio Imager 2 (Zeiss).

### NO measurement assay (Griess)

The endogenous NO concentration was measured indirectly from the nitrite content using the Griess reagent, according to Green and collaborators^55^. Adult specimens and embryos at different developmental stages were homogenized in PBS and centrifuged at 20000 g for 30 min at 4 °C. Total protein concentration was determined by the Bradford assay using a Bio-Rad Protein Assay Reagent (Bio-Rad), bovine serum albumin was used as a standard. The supernatant of each sample was then analysed for nitrite content using a spectrophotometer (λ = 540) and reported as nmol of nitrite per mg of protein. The experiment was performed on biological triplicates for each sample.

### *In vivo* NO modulation assays with L-NAME and TRIM

We decreased the NO production during amphioxus development using two types of drugs that alter the NOS activity: an analog of arginine, Nω-Nitro-L-arginine methyl ester hydrochloride (L-NAME, Sigma Aldrich, stock solution in filtered sea water, FSW) and 1-(α,α,α-trifluoro-o-tolyl)-Imidazole (TRIM, Cayman Chemical, stock solution in DMSO) which interferes with binding of both L-arginine and tetrahydrobiopterin to their respective sites on the NOS enzymes. Untreated control larvae were raised in FSW. Additional controls included: inactive enantiomer Nω-Nitro-D-arginine methyl ester hydrochloride (D-NAME, Sigma Aldrich) for L-NAME, and DMSO for TRIM. The treatments were started and blocked at different developmental stages and the phenotype was always observed at 3 dpf larvae (Suppl. Fig. S2). All the experiments shown in Suppl. Fig. S2 were performed with 100 µM, 1 mM and 10 mM L-NAME at 18 °C as pilot experiments. We repeated the *in vivo* experiments adding the TRIM at the neurula stage, which proved to be the most sensitive stage to drug treatment. Therefore, 24 hpf embryos (neurula stage) were treated with 50, 75 and 100 µM TRIM for the time periods indicated in Suppl. Fig. S2. Larvae at 72 hpf were fixed in 4% PFA, dehydrated and stored in 70% ethanol, and the morphology was initially analysed using a stereoscope and then, for image acquisition, using a Zeiss EVO MA LS Scanning Electron Microscope.

### *In vivo* NO localization assay

NO localization was performed using 4-amino-5-methylamino-2′,7′-difluorofluorescein diacetate (DAF-FM-DA), the most sensitive cell permeable and non-fluorescent reagent that combines with NO forming benzotriazole, a fluorescent compound^56^. Embryos at different developmental stages were incubated for 20 min in the dark with 5 μM DAF-FM-DA in FSW. After treatment the animals were washed and incubated in FSW for 30 min and quickly fixed in 4% PFA. The fluorescence was visualised with ZEISS Axio Imager Z1 fluorescence microscope equipped with a λEXC = 470 ± 40, λEM = 525 ± 50 filter.

## Electronic supplementary material

Supplementary Figures
