# Supplementary material for: Nitric Oxide regulates mouth development in amphioxus
Source: Sci Rep. 2017 Aug 16;7:8432. doi: 10.1038/s41598-017-08157-w (PMC5559612; doi:10.1038/s41598-017-08157-w)
Supplement: Supplementary file 1 — Supplementary Figures [file 41598_2017_8157_MOESM1_ESM.pdf]

Nitric Oxide regulates mouth development in amphioxus

Giovanni Annona[1], Filomena Caccavale[1], Juan Pascual-Anaya[2],
Shigeru Kuratani[2], Pasquale De Luca[3], Anna Palumbo[1], Salvatore D'Aniello[1,*]

[1] Biology and Evolution of Marine Organisms, Stazione Zoologica Anton Dohrn di Napoli, Villa Comunale 1, 80121, Napoli, Italy

[2] Evolutionary Morphology Laboratory, RIKEN, Minatojima-minami 2-2-3, 650-0047 Kobe, Hyogo, Japan

[3] RIMAR, Stazione Zoologica Anton Dohrn di Napoli, Villa Comunale 1, 80121, Napoli, Italy

[*]**Corresponding Author:** Salvatore D'Aniello < salvatore.daniello@szn.it >

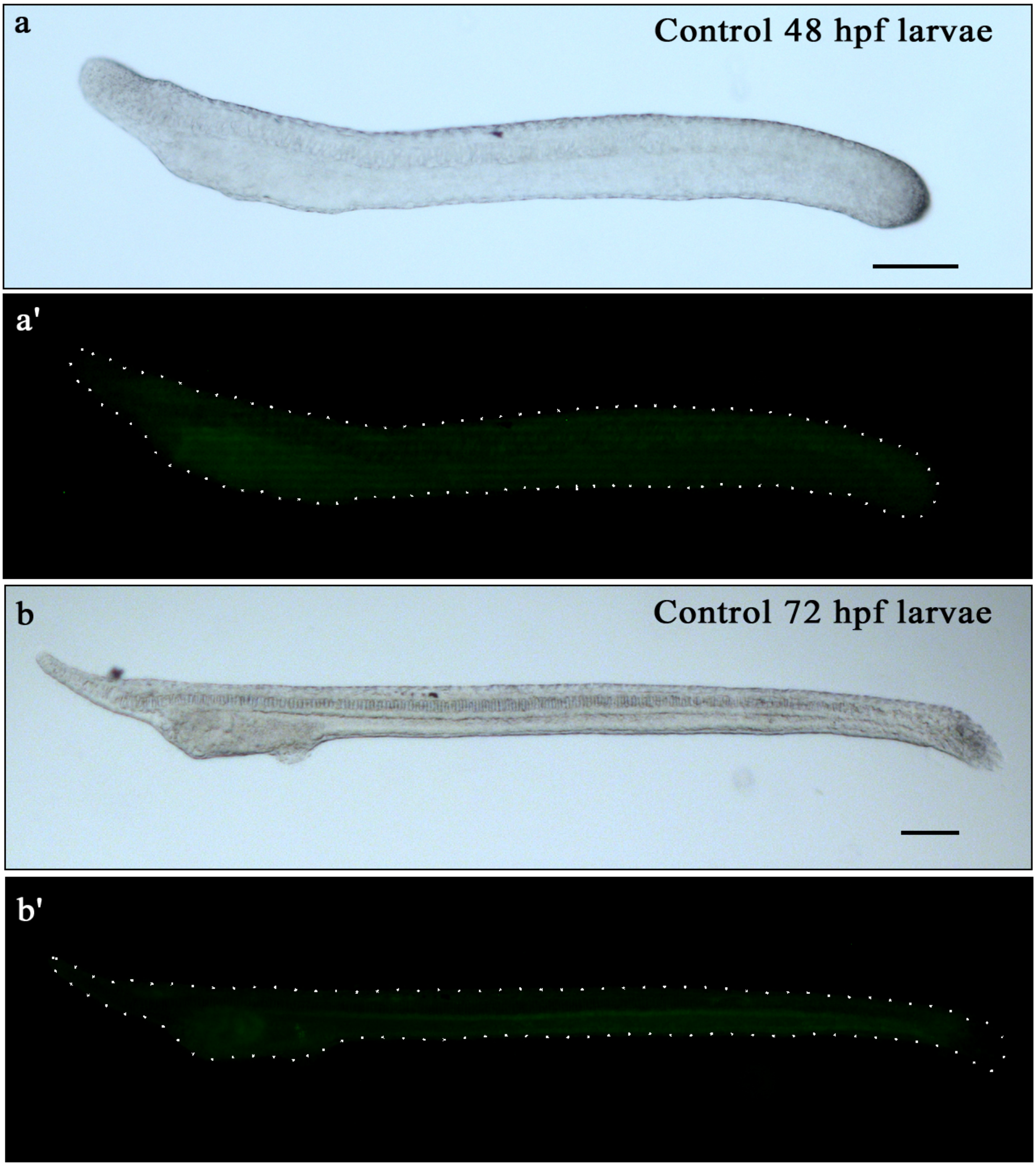

Supplementary Figure S1:
Endogenous green fluorescence emission in not treated *B. lanceolatum* larvae (negative control), showing a negligible non-specific signal at the same laser intensity as used for DAF-FM-DA experiments. a) pre-mouth larvae (48 hpf) in bright field, a') the same specimen in fluorescence. b) 72 hpf larvae in bright field, b') the same specimen in fluorescence. Rostral part is on the left, dorsal part on the top.Scale bars: 50 µm.

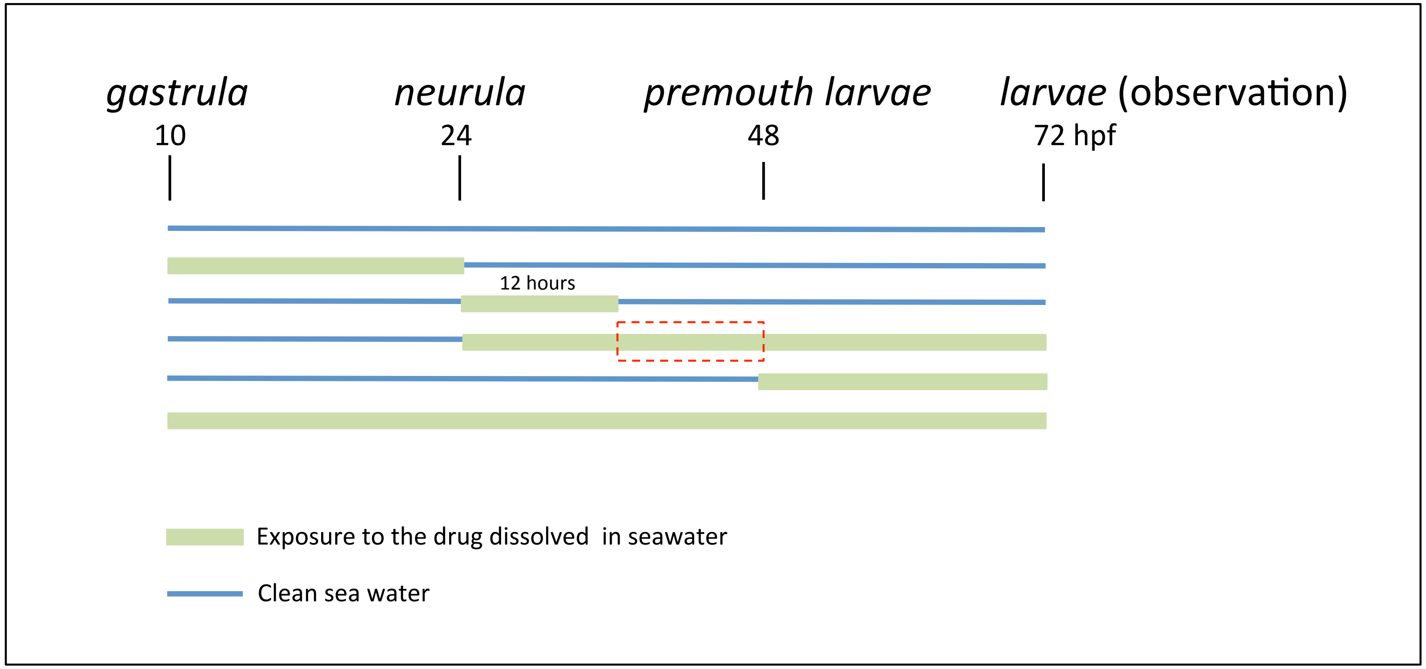

Supplementary Figure S2: Scheme of drug treatments

The drug incubation period, from gastrula to larvae, is schematized by green bars, while blue thin bars indicate that the embryos are in FSW. Several washes with sea water are were performed to end treatments. All samples were observed at 72 hpf for the general morphology (body plan shape, length) and in particular for the pharyngeal area development.

Supplementary Figure S3

Protein and coding sequences (CDS) of the *B. lanceolatum Nos* genes, extracted from the draft genome sequence. See Materials and Methods for details.

>*B. lanceolatum* NOSA, protein

MEGQQNIIVVGLVKREVDGLGVSAKRREVPGHSAVVIEEVIRGGPADHTGMVQPGDTIVAVN
GQDIEGLSYQEATKVIRDIEPGKPVELVLRGPKWFHTYLETVRLSTGETKTVRVTRPNGPIG
ALTLLIQRMRGNTFDKRAIRKLENRGDIPLYNRGVLMNGDVIKPIPNGTVSNGCPVTGHRAA
TVGPSRTRRPVRLKNWLNERRINDNLHSKANDLNPCNDGRCLGPLMRPRPFTAPGYFRPKEE
VLEQAKKFIFEYYASLKSSDAGERDRRWRDVQVQVEEKGVYHLTYDELLYGAKMSWRNAPRC
IGRIQWSKLQLFDARDVTTARGMFEAICRHIKWCTNGGNIRSAITVFPPRTDGRHDYKVWNG
QFLKYAAYQLPDGSILGDPINLEFTEVCQALGWKGEGTRFDLLPMVLSANGEDPEWFDLPKD
IVMEVNITHPKYDWFEELGIKWYCVPAVSNMLFDCGGLEFSAAPFSGWYMGTEVGRDLCDEN
RLNITEAVGKRMGLDVARNSSLWKDAVFVEVNIAVLHSFQRNNATIMDHHTASETFMKHLEN
EQRLRGGCPSDWVWIVPPMSASLTEVFHQEMANYHVRPSFEYQEDAWLLHLWRKKPEHPVLL
KYAYDENPKRKYRLKEVALAVKFAAVLMKKALNKRVKTTILYATETGKSESFASSTLTIFKH
AFDAKMMCMDEYDITNLEKEELVIVVTSTFGNGDPPDNGEAFGQALLHMRHPPGSDKKRSGS
VRRVSSISKASYRRQQKLLRQLRDGGALGKVRYGVFALGSRAYPHFCAFGHAIDTLFESLGA
ERIHPVGEGDELCGQEESFRVWVKGAFKSACERYDVGHYVNMEEANASLLGSDFSWAPGKFR
VLQTKGLPETDILEGLSKLHRRTVVSSTVISRTQLQAPESSRQTCLVQLETHGAQELRYVPG
DHVAVFPANEDRLVQAILDRVEKGTNPDAVIQIEALQEKKIGAGLVKSWTPHDRLPTCSLRT
ALSRYLDVTTPPSPQLLLYLAMHATSSRERAELEALGKGGLRYEDWKFEAAPTLPEVLQHYP
SLQVPPALLLSQLPVLQQRYYSISSSPHMYPGQIHATVAVVKYRTRGGQGPEHSGVCSNWLN
TIKSNESVPCFIRTAKNFHLPENSSLPVLMVGPGTGIAPLRSFWQQRQVDIKAGTTSRHPPG
DMTLVFGCRQSRVDHIYKEETAQARRDGALTDLYIALSREPGTTKTYVQDIIRQQIPKKVLD
LVLKDGGHIYVCGDVTMATDVGETVQRILVEHGGMSVARAEDFINNMKDNNRYHEDIFGVTL
KTHEVEDAARKRSPSFISLSEENTSL

>*B. lanceolatum* NosA, CDS

ATGGAGGGACAACAGAACATCATCGTCGTCGGACTGGTCAAGAGGGAGGTTGATGGACTGGG
AGTATCGGCGAAGCGACGGGAAGTACCCGGTCATTCTGCGGTGGTCATCGAGGAGGTGATCC
GAGGCGGGCCTGCTGACCACACCGGGATGGTGCAGCCAGGGGACACCATCGTCGCTGTAAAC
GGGCAGGACATCGAGGGGTTGTCGTACCAGGAGGCGACCAAGGTCATCAGGGACATCGAGCC
GGGGAAGCCGGTCGAGCTCGTCCTCCGCGGACCGAAGTGGTTCCACACCTACCTGGAGACCG
TCCGTCTGAGCACCGGGGAGACCAAAACCGTGCGAGTCACCCGGCCTAACGGGCCCATCGGC
GCCCTGACACTCCTCATACAGCGCATGAGAGGAAACACTTTTGACAAGAGAGCCATACGTAA
ACTTGAGAATCGCGGCGACATCCCGCTCTACAATCGCGGCGTACTGATGAACGGTGATGTCA
TCAAGCCCATCCCGAACGGTACCGTATCGAACGGCTGTCCGGTCACTGGGCACAGGGCAGCC
ACGGTGGGACCGTCCAGGACCAGGAGGCCAGTGCGACTGAAGAACTGGCTGAACGAAAGGAG
AATCAATGACAACCTCCACTCGAAGGCAAACGACTTGAACCCGTGCAATGACGGCAGATGTC
TCGGTCCGCTGATGCGACCACGACCGTTCACAGCACCCGGCTACTTCCGGCCGAAGGAAGAA
GTCCTCGAACAAGCAAAGAAGTTCATCTTCGAGTACTACGCTTCCTTGAAGAGTTCGGATGC
TGGTGAGCGTGACAGACGTTGGAGAGACGTCCAGGTGCAGGTGGAGGAGAAAGGTGTGTACC
ACCTGACGTACGACGAGCTGTTGTACGGCGCCAAGATGTCGTGGAGGAATGCGCCAAGATGC
ATCGGACGTATCCAGTGGTCCAAACTGCAGCTGTTTGACGCCCGTGACGTCACAACGGCCAG
AGGAATGTTTGAGGCTATCTGCCGACACATCAAATGGTGCACCAACGGCGGGAATATCAGGT
CTGCCATTACTGTTTTCCCGCCACGCACGGACGGTCGCCATGACTACAAGGTGTGGAACGGA
CAGTTCCTGAAATATGCCGCCTACCAGCTGCCTGACGGGTCCATCCTCGGCGATCCCATCAA
CCTAGAATTCACAGAGGTATGCCAGGCTCTGGGCTGGAAGGGGGAGGGCACACGGTTCGACC

TGCTGCCCATGGTTCTGTCTGCTAACGGGGAGGACCCGGAGTGGTTCGACCTCCCGAAGGAC
ATCGTGATGGAGGTCAACATCACACATCCCAAGTACGACTGGTTTGAAGAGTTGGGCATCAA
GTGGTACTGTGTCCCTGCCGTGTCCAACATGTTGTTTGACTGCGGCGGTCTGGAGTTTTCAG
CGGCTCCCTTCAGCGGCTGGTACATGGGGACGGAGGTGGGCAGGGACCTGTGTGACGAGAAC
AGGCTCAACATCACAGAAGCAGTAGGAAAGAGGATGGGTCTGGACGTCGCAAGAAACTCCTC
CCTCTGGAAAGACGCCGTTTTCGTGGAGGTCAACATCGCCGTCTTGCATAGCTTCCAGCGCA
ACAACGCCACGATTATGGATCACCACACGGCTTCGGAGACCTTCATGAAACACCTGGAGAAC
GAACAGCGCCTGCGCGGGGGATGCCCTTCTGATTGGGTGTGGATCGTGCCCCCAATGTCGGC
GTCTCTCACCGAGGTTTTCCATCAAGAGATGGCGAACTACCATGTGAGGCCGTCCTTCGAAT
ATCAGGAGGACGCCTGGCTGCTTCACCTCTGGAGAAAGAAGCCGGAGCACCCAGTCCTTCTG
AAGTACGCCTACGATGAAAACCCCAAAAGGAAGTACCGTCTGAAGGAGGTGGCTCTGGCGGT
AAAGTTCGCTGCCGTGCTGATGAAGAAAGCTCTCAACAAGCGAGTAAAGACCACCATCCTGT
ACGCCACAGAAACCGGCAAGTCGGAATCATTCGCATCTTCTACACTCACGATCTTCAAACAC
GCGTTTGACGCAAAGATGATGTGTATGGATGAGTACGACATAACAAACCTGGAGAAGGAGGA
ACTCGTCATCGTCGTCACCAGTACCTTCGGGAACGGAGACCCGCCGGACAACGGAGAGGCTT
TTGGTCAGGCTTTACTCCACATGAGGCATCCACCTGGAAGTGATAAAAAGAGATCCGGCAGT
GTCCGGAGGGTGAGCAGCATCTCCAAGGCCTCCTACCGACGTCAGCAGAAACTGCTGCGGCA
GCTCCGGGACGGCGGGGCGCTCGGCAAAGTCAGATACGGTGTGTTTGCCCTCGGGTCCCGCG
CCTATCCACACTTCTGCGCCTTCGGCCACGCCATCGACACGCTGTTTGAGTCGCTGGGAGCG
GAACGGATCCATCCTGTGGGTGAAGGAGACGAGCTGTGTGGACAGGAGGAGTCCTTCCGGGT
CTGGGTCAAGGGCGCCTTTAAGTCTGCATGTGAGAGGTACGATGTCGGCCATTACGTGAACA
TGGAGGAGGCGAACGCGTCCCTTCTCGGGAGTGACTTCAGCTGGGCGCCGGGGAAGTTCCGT
GTCCTACAGACCAAGGGACTGCCGGAGACAGACATCCTGGAGGGTCTGTCCAAACTGCATCG
CAGAACAGTTGTGTCCAGTACAGTCATCTCCAGAACTCAGCTACAAGCTCCGGAGTCCAGCC
GACAGACCTGCCTGGTGCAGTTGGAAACTCACGGGGCCCAGGAGCTGAGGTATGTTCCCGGG
GATCACGTGGCCGTCTTCCCAGCCAATGAGGACCGACTAGTGCAGGCGATTCTGGACAGAGT
GGAGAAGGAACAAACCCCGACGCCGTCATTCAGATAGAAGCGCTGCAAGAGAAAAAAATTG
GAGCTGGTTTGGTCAAATCATGGACGCCCCACGACCGACTGCCGACATGTTCTTTGCGCACG
GCACTATCCCGTTATCTTGACGTCACCACCCCACCGTCACCACAGCTGCTCCTCTACCTGGC
GATGCACGCGACGTCAAGCAGGGAGAGAGCTGAACTCGAGGCTTTGGGCAAGGGAGGACTGA
GGTACGAGGACTGGAAGTTTGAGGCGGCCCCGACTCTGCCCGAGGTGCTGCAGCACTACCCG
TCCCTGCAGGTCCCGCCCGCCCTGCTGCTCAGTCAGCTGCCGGTCCTGCAGCAGCGCTACTA
CTCCATCAGCTCCTCCCCACACATGTACCCGGGCCAGATCCACGCTACTGTAGCCGTGGTCA
AGTACAGGACGCGTGGTGGCCAGGGTCCTGAGCACAGCGGTGTGTGTTCTAACTGGCTCAAC
ACCATCAAGAGCAACGAGTCCGTGCCTTGCTTCATTCGCACGGCCAAGAATTTCCACCTTCC
TGAGAACTCCTCCCTCCCTGTCCTGATGGTAGGTCCCGGGACTGGCATCGCCCCTCTCAGGA
GCTTCTGGCAGCAGAGACAGGTGGACATCAAGGCTGGTACCACGTCCAGACATCCTCCAGGT
GACATGACCCTGGTGTTCGGCTGCAGACAGTCCAGGGTGGACCACATCTACAAGGAGGAGAC
GGCGCAGGCAAGGAGAGACGGAGCCCTGACAGACCTCTACATAGCACTGTCCAGGGAACCTG
GGACAACTAAGACCTACGTCCAAGACATCATACGTCAGCAAATTCCCAAGAAGGTGCTGGAC
CTGGTTCTGAAAGATGGCGGACACATCTACGTGTGTGGTGACGTCACCATGGCCACTGACGT
CGGCGAGACCGTTCAGCGAATCCTGGTGGAGCACGGCGGGATGTCTGTCGCCAGGGCAGAGG
ACTTCATCAACAACATGAAGGACAACAACCGTTACCACGAGGACATCTTCGGTGTGACACTG
AAGACACACGAGGTTGAGGATGCAGCTCGCAAGAGAAGTCCGTCTTTCATCAGTCTTTCGGA
AGAGAACACTTCTTTGTGA

>*B. lanceolatum* NOSB, protein

MPPVQSRETNGTNPNVTPNGCPFTGSTSTAPRRAQILTDHLRNKHLTDTLHLKATNVAPCSS
GKCMGSVVRQREPFPPGYKRPKEEVLEQAKEFIDDYYASMKKENSAEHERRWIDIQAQVEEK
GLYDLEYDELLYGAKMAWRNASRCIGRYQWNNLQVIDYRNVKTAQEMFDAACDHIRYATKDG
QMKTAISIFPMRTDGKHDYKFWNKYLFQYAGYQQPDGSVIGDPANVELTEVCQSLGWKGKGT
PYDFLPAIVSANGEDPVIFEWPEDVPLELELRHPKHDWFEELAIKVNCVPLQGDMLFDVGGM
QFPACPITGWFQSTEPVRDLLDENRYNMAEIIAKKLGYDTSRYNSLWKDAAFLEVHIAVIHS
FQMKGVMTQDPYTLTESFMKHMEREHRVRGGCPSDWVWIVPPSCSSLTPVFHQEMLNYHLRP
ALEYQEAGWKIHTWKTPPTAPIFTQYIRTKKRCSFKAAAMAVMFASGLMRKTLNKRVKSTIL
FASQTGKAETFSNSLCDLYKHAFDAKVVCMDEYDMAQLDKEQLVVIVTSTFGNGDPPDNGET
FARALMQMKNQNGQASPRDSPLSSVRFAVFALGSRAYPHFCAFGHSVDTLLEQLGARRVHAV
GEGDELCGQEESFRAWAESAFKSACLSYDVGHGVDMNEATANLLGSDLSWAPDKFRLVQAKT
KTDTDILQGLSTVHRKNIVPCTFISRTQLQAPDSSRQTILVRLDTKDNEELEFTPGDHLGVF
PANEDHLVQAILDRIEGGTKPDDVLEIEALHKKLTAAVVTKTWMPFERLPPCSLRTALSRYL
DITTPPSPQLLGSLSMHTSAPSERAELEELAKGGTKYENWKFERAPTLPEVLQEYPSLQVPP
ALLLSQLPVLQQRYYSISSSPHMYPGEIHATLAVVSYRSQGGKGPVHNGVCSSWFNRLEEGE
TVPCFVRAAKNFHLPADPSMPVLMVGPGTGIAPLRSFWQQRQMDIKSGNHGDMTLVFGCRQS
QIDHIYKDETAQAKTDGALTDIYTAYSREPDTPKTYVQHVLYNQLSERVKELLKNNGHIYVC
GDVAMADDVCTTVQKILEEKSAMTSTESEALIRMLKDSNRYHEDIFGVTLNTKEVREAARSV
ESAESNAK

>*B. lanceolatum* NosB, CDS
ATGCCACCGGTACAATCCAGAGAAACGAACGGCACCAATCCTAATGTCACCCCGAACGGGTG
TCCCTTTACGGGGTCTACCAGCACAGCTCCGCGGAGAGCCCAGATTCTGACAGACCACCTGC
GGAACAAGCACTTGACCGATACTTTACACCTGAAGGCAACCAATGTAGCCCCTTGCAGTTCA
GGAAAATGCATGGGCTCGGTGGTTCGGCAGAGAGAGCCTTTTCCCCCGGGTTACAAGAGGCC
GAAGGAAGAAGTGCTGGAACAGGCGAAGGAATTTATCGACGACTACTACGCTTCGATGAAAA
AGGAAAACAGTGCAGAACATGAGAGGCGGTGGATTGACATACAAGCCCAGGTCGAAGAGAAA
GGTTTGTACGACTTGGAGTACGATGAGCTTCTGTATGGCGCCAAAATGGCCTGGCGGAACGC
ATCCAGATGTATCGGACGGTACCAATGGAACAACTTACAAGTGATTGACTATCGCAACGTCA
AGACGGCCCAAGAAATGTTTGACGCCGCCTGCGATCATATTAGGTACGCCACCAAGGATGGG
CAGATGAAGACCGCCATCAGCATTTTCCCAATGCGCACAGATGGAAAACACGACTACAAGTT
CTGGAATAAGTACCTGTTCCAGTACGCTGGGTACCAGCAGCCTGACGGTTCAGTCATTGGAG
ATCCCGCCAACGTGGAACTTACCGAGGTGTGCCAAAGTCTCGGCTGGAAGGGCAAAGGAACT
CCATATGATTTTCTACCCGCCATTGTCTCTGCTAATGGCGAGGACCCGGTCATCTTCGAATG
GCCAGAAGACGTACCCCTGGAACTGGAACTGCGGCATCCAAAGCACGACTGGTTCGAAGAGC
TGGCCATAAAGGTGAACTGCGTACCGCTGCAAGGAGACATGCTATTCGACGTTGGCGGGATG
CAGTTTCCTGCCTGTCCAATCACAGGCTGGTTTCAGAGCACGGAGCCTGTGCGCGATCTGTT
GGACGAAAACAGATACAACATGGCAGAGATCATCGCCAAGAAGTTGGGATATGACACTTCCC
GGTACAACTCGCTGTGGAAGGATGCTGCCTTCCTGGAGGTTCACATTGCAGTTATACACTCT
TTTCAGATGAAGGGAGTGATGACCCAGGATCCGTACACCCTGACCGAGAGCTTCATGAAGCA
CATGGAGAGGGAACACCGTGTCCGCGGCGGGTGTCCGTCCGACTGGGTCTGGATCGTTCCTC
CCTCCTGCTCCTCTCTCACTCCCGTCTTCCACCAGGAGATGCTGAACTACCACCTCAGGCCC
GCTCTGGAGTACCAGGAGGCGGGATGGAAGATACATACATGGAAGACTCCTCCGACTGCCCC
CATCTTCACACAGTATATCCGCACAAAGAAGCGCTGCAGTTTCAAGGCGGCAGCGATGGCTG
TGATGTTCGCTTCAGGGCTGATGAGAAAGACTCTGAACAAGAGAGTGAAGTCCACGATCCTC
TTCGCCTCTCAGACCGGAAAGGCAGAGACATTCTCCAACTCACTGTGCGACCTCTACAAGCA
TGCATTCGATGCAAAGGTGGTGTGTATGGATGAGTATGACATGGCGCAACTCGACAAAGAGC
AACTGGTCGTCATAGTGACAAGCACTTTTGGAAACGGGGATCCCCCGGACAATGGCGAGACC
TTCGCTCGTGCTTTAATGCAAATGAAGAATCAGAATGGTCAAGCAAGTCCAAGAGACAGCCC
ACTGAGCTCTGTCAGGTTTGCGGTGTTCGCCCTTGGATCCCGTGCCTACCCGCACTTCTGTG
CCTTTGGCCACAGCGTGGACACCCTCCTGGAACAGCTGGGGGCTCGGCGGGTCCACGCCGTG

GGCGAAGGGGACGAACTGTGCGGCCAGGAGGAGTCATTCCGCGCATGGGCAGAGAGTGCATT
CAAGTCTGCCTGTTTGAGTTACGATGTTGGGCACGGTGTCGACATGAACGAAGCCACTGCCA
ATCTGCTGGGCAGCGATCTGAGTTGGGCGCCGGACAAGTTCCGACTTGTACAGGCCAAGACT
AAGACGGACACTGACATTCTACAAGGTTTGTCCACTGTGCATCGTAAGAATATTGTGCCATG
TACCTTCATTTCGCGGACCCAACTGCAGGCACCGGACTCAAGTCGACAAACCATCTTGGTTC
GACTGGATACGAAGGACAACGAAGAGTTGGAGTTTACTCCCGGCGATCACCTCGGCGTCTTC
CCGGCCAATGAAGACCACCTTGTGCAGGCGATCTTAGATAGAATCGAGGGTGGAACCAAACC
GGATGATGTGCTGGAGATAGAAGCGCTGCACAAGAAGCTGACAGCAGCTGTGGTGACGAAAA
CGTGGATGCCTTTCGAGCGCTTGCCGCCATGTTCTTTGCGCACGGCACTCTCCCGGTACCTA
GACATCACTACCCCACCATCGCCGCAGCTGTTGGGTAGTCTGTCCATGCACACCAGCGCTCC
ATCAGAGCGAGCAGAGCTGGAGGAACTCGCTAAGGGTGGTACAAAGTACGAGAACTGGAAGT
TCGAGCGCGCCCCTACCCTACCGGAGGTTCTGCAGGAGTACCCGTCCCTACAGGTTCCGCCC
GCCCTGCTGCTGAGTCAGCTGCCGGTCCTCCAGCAGCGCTACTACTCCATCAGCTCCTCCCC
ACACATGTACCCGGGAGAGATCCACGCTACTTTAGCTGTGGTCAGCTACCGTTCTCAGGGCG
GAAAGGGTCCGGTACACAACGGCGTTTGCTCCTCGTGGTTTAACAGGCTTGAGGAGGGTGAA
ACGGTCCCCTGCTTCGTCAGAGCGGCGAAGAATTTCCACCTCCCTGCCGACCCCTCCATGCC
TGTGCTGATGGTCGGCCCCGGTACGGGCATCGCCCCGCTGCGCAGCTTCTGGCAGCAACGCC
AGATGGACATCAAGTCCGGTAACCATGGTGACATGACGCTGGTGTTCGGATGTCGACAGTCC
CAGATAGACCACATCTACAAGGACGAGACGGCCCAGGCCAAGACAGACGGAGCGCTGACGGA
CATTTATACAGCCTACTCCAGAGAACCGGACACACCAAAGACCTACGTCCAGCATGTTTTGT
ACAACCAACTCTCCGAGAGAGTGAAAGAACTCCTGAAGAATAATGGACATATCTATGTCTGT
GGTGACGTAGCCATGGCTGATGACGTATGCACCACCGTGCAGAAAATACTGGAAGAAAAGTC
CGCGATGACGTCGACGGAGAGCGAAGCATTAATCAGAATGTTGAAGGACTCCAATCGCTATC
ACGAGGACATCTTTGGGGTGACTCTCAACACAAAGGAGGTTCGAGAGGCAGCTCGAAGCGTC
GAGTCAGCCGAGAGCAATGCAAAATGA

>*B. lanceolatum* NOSC, protein
MEIRPNVIAVKLMKREGDGLGFLVKQRSCNPPVIVSDVVRGGAADQSGLIQVGDLILSVDGT
SLETVPYSDALQVLRAVEVGKPTEIILRGPEGFATKLETTFTGTGIPKTVRITTAESPLRSL
ALSPARRLIKRITGNSHVKSIDHINAEALKEKETVSNGPLGTDHSGRTMETPKTCSSIAVQT
SPEEPPTKEVNGQCNGNATVAQLDKTKLTLDTISIALKRESVNGNELGRPIESGPSSRRNSA
TLSPSAKPRFARMKNWMNDKQMTDTLHNKATPINPCSGTKCLGSLMRPNAAAQAKSARPAGE
PRPKDEVLEHAKEFLDEFFASIKRANTQAHKQRWAEAKVQIEEKGWYELTQMELTYGAKLAW
RNAPRCVGRIQWSKLQVFDARYVTTARGMYEAICNHIKYATNKGNLRSAITIFPARTDGKHD
FKVWNSQFVRYAGYKQPDGSVVGDPASVEFTEICQSLGWKGKNGPFDVLPMVLSANGQDPEL
FELSRDLVLEVELKHPKYAWFKELGMKWYALPAVANMLFDVGGVEFPAAPFSGWYMCTEIGR
DLCDINRYNYTEQIAKRMGLDTGRASSLWRDLAMIEANVAILHSFQTGNVTITDHHTACESF
MKHMENEQRLRGGCPADWVWIVPPVSASLTPVFHQEMISYYLRPSYEYQEDAWKTHVWKKKE
DTKRIMPGKAKRKFGFKEVAKAVKFSAKLMGKALAKRVKATILYATETGKSERYAKTVCEIF
KHAFDAKVMCMDDYDIMHLEHETLVIVVTSTFGNGDPPDNGESFGQALLEMRHPPMDNNDNR
PPIRRVSESSSDGGHTKRLSSISSSATERRRKFSHQMKERDMDSMDLDGGPLSNVRFSAFGL
GSRAYPGFCAFAHAVDTLFGELGGERIYKMGEGDELCGQEESFRKWAKGVFKAACDTFCVGD
DLNMSEASSTLLNSDATWSPDKFRLIPAEGVKEFEIWEGLSKVHHRNVVPCRLISRENLQAP
DSGRETILVRLDSQGSDDLNYVPGDHLAVFPANEDHLVQAVLDRLDNAPDPDSIVNMEVLQE
KQTPLGAIRTWMTSERLPPCSLRTALSRYFDITTPPSPQLLQHLATQATDEEEKKELEVLGK
GDARYEDWKFERTPNLVEVLEDYPSLKVAPTLLLSQLPFLQQRYYSISSSQRMYPGEIHATV
AVVRFATQGGVGPTHNGVCSSWLNRIEKDDIVPCFVRGAQNFHLPEDPTVPLMMVGPGTGIA
PFRSFWQHRQMEVTSGDPHHRPKYGQMTLVFGCRQSKMDDIYKHETAQAKEDGALTEVYTAL
SREPGVPKSYVQNVILDLIPEKVCDLLMKHNGHFYVCGDVSMAADVCNTLEKAMEKQQGMTP

NKAKDFVDKLKDCNRYHEDIFGVTLRTQEVTDRVRSAARKNWVRVKRLRPSTVVPPTRGLSP
VRESSSLSSTPMSTPSATPVNSPYSSPLHRPDPTVCANGDLPSLHIEE

>*B. lanceolatum* NosC, CDS

ATGGAGATTCGGCCTAACGTGATTGCCGTCAAGTTGATGAAGAGGGAGGGGGATGGACTAGG
CTTTCTGGTGAAGCAGCGCTCCTGCAACCCTCCAGTCATCGTCTCTGACGTGGTGAGAGGCG
GGGCGGCAGACCAAAGCGGGTTGATCCAAGTCGGCGACCTTATCCTCTCCGTCGACGGCACG
AGTTTGGAGACCGTGCCCTACAGCGACGCTCTACAGGTTCTCAGGGCGGTGGAGGTGGGAAA
ACCGACGGAGATCATCCTCCGCGGGCCCGAGGGTTTTGCGACGAAGCTCGAGACCACCTTCA
CGGGGACTGGCATCCCGAAAACGGTCCGAATAACCACTGCGGAAAGCCCTCTGCGCTCTCTT
GCCCTCTCGCCGGCTCGCAGGCTCATCAAGAGGATAACCGGCAACTCTCACGTCAAATCAAT
CGACCACATCAACGCAGAAGCGCTCAAAGAGAAAGAAACTGTTTCTAATGGACCGCTAGGCA
CTGACCACAGCGGCCGAACAATGGAGACGCCCAAGACGTGTTCGTCTATCGCTGTACAGACA
TCACCAGAAGAGCCCCCCACCAAAGAAGTCAACGGACAGTGTAACGGCAATGCCACAGTTGC
ACAGCTAGACAAGACAAAACTGACTCTAGACACGATTTCTATCGCCTTGAAGAGGGAGAGCG
TCAACGGGAATGAGCTGGGAAGGCCCATCGAGTCGGGGCCGTCTTCCCGGCGGAACTCGGCC
ACGCTGAGCCCGTCCGCAAAACCGAGGTTTGCCCGCATGAAGAACTGGATGAACGACAAGCA
GATGACAGACACCCTGCACAACAAAGCTACACCGATAAACCCCTGTTCGGGTACAAAGTGTC
TGGGCTCGTTGATGCGTCCTAACGCCGCTGCCCAGGCCAAGAGCGCCCGGCCGGCTGGGGAA
CCCAGACCGAAGGATGAAGTCTTAGAGCACGCCAAAGAGTTCCTGGACGAATTTTTCGCGTC
AATCAAACGCGCTAACACACAGGCACACAAGCAGAGATGGGCGGAGGCGAAGGTGCAGATCG
AGGAGAAGGGTTGGTACGAGCTGACGCAGATGGAGCTAACGTACGGAGCCAAGCTCGCCTGG
AGGAACGCGCCCAGATGTGTGGGCAGAATACAGTGGTCTAAACTACAGGTGTTCGACGCTCG
GTACGTGACTACAGCACGAGGCATGTATGAAGCCATTTGCAACCATATCAAGTATGCAACCA
ACAAGGGAAACCTCAGGTCGGCCATCACTATCTTCCCCGCTCGCACCGATGGCAAACACGAC
TTCAAGGTGTGGAACTCGCAGTTTGTCCGGTATGCCGGGTACAAGCAGCCGGACGGTTCCGT
CGTTGGTGACCCAGCAAGCGTGGAGTTCACTGAGATCTGCCAGAGCCTAGGATGGAAAGGGA
AGAACGGACCGTTTGATGTCCTGCCCATGGTGCTGTCTGCTAACGGTCAGGACCCGGAGCTG
TTTGAGCTGTCAAGGGACCTAGTGTTGGAGGTGGAGCTCAAACATCCCAAATACGCGTGGTT
CAAAGAGCTTGGAATGAAGTGGTACGCCCTGCCGGCGGTGGCTAACATGCTGTTCGACGTGG
GCGGAGTCGAGTTCCCGGCAGCGCCCTTCAGCGGCTGGTACATGTGCACGGAGATCGGCAGA
GACCTGTGCGACATCAACAGATACAACTACACAGAGCAAATTGCCAAAAGGATGGGGCTTGA
TACAGGACGAGCGTCGTCACTATGGAGAGACCTGGCTATGATCGAGGCAAATGTCGCCATTC
TCCATAGCTTTCAGACAGGCAATGTGACCATCACCGATCACCACACCGCGTGTGAGTCCTTC
ATGAAGCACATGGAGAACGAACAGCGCCTGCGCGGTGGGTGTCCGGCGGACTGGGTCTGGAT
CGTCCCGCCTGTCTCTGCTTCTCTCACTCCCGTCTTCCACCAGGAGATGATCAGCTACTACC
TGAGACCGTCTTACGAATATCAGGAGGATGCGTGGAAGACCCACGTGTGGAAGAAGAAGGAG
GACACCAAGAGGATCATGCCTGGGAAGGCGAAAAGAAAGTTCGGCTTCAAGGAAGTGGCCAA
AGCCGTGAAGTTCTCTGCAAAGCTGATGGGAAAAGCGCTGGCTAAGCGGGTGAAGGCGACCA
TTCTGTACGCTACAGAGACCGGCAAGTCGGAGCGGTACGCCAAGACGGTCTGTGAAATCTTC
AAGCACGCGTTTGATGCCAAGGTGATGTGTATGGATGATTATGACATCATGCACCTGGAACA
TGAGACACTGGTCATCGTGGTGACCAGCACTTTCGGCAACGGTGACCCACCGGACAATGGGG
AGTCGTTTGGCCAAGCCCTTCTGGAGATGCGACATCCACCAATGGACAACAACGACAACAGA
CCCCCTATACGGCGGGTTTCTGAATCAAGCAGTGACGGTGGGCACACGAAGCGGCTGAGCAG
CATTTCCAGCAGCGCCACCGAGCGGCGCCGGAAGTTCTCGCACCAGATGAAGGAGCGGGACA
TGGACAGCATGGACCTGGACGGCGGGCCCCTCAGTAATGTCAGGTTTTCGGCCTTTGGGCTT
GGTTCTCGGGCCTACCCAGGATTCTGCGCGTTTGCGCATGCGGTTGACACCCTGTTCGGTGA
GCTGGGAGGAGAACGGATCTACAAGATGGGGGAGGGGGACGAGCTGTGCGGACAGGAGGAGT
CCTTCCGAAAGTGGGCTAAAGGCGTGTTCAAGGCGGCCTGTGATACTTTCTGTGTCGGTGAC
GACCTAAACATGAGCGAGGCTAGCTCCACCCTGCTGAATTCGGACGCAACCTGGTCACCGGA
CAAGTTCCGACTCATTCCGGCAGAGGGCGTTAAGGAGTTTGAAATCTGGGAAGGACTATCGA

AGGTGCACCATAGAAACGTGGTACCCTGCAGACTCATCTCCAGAGAAAACCTGCAAGCACCC
GACTCTGGCCGGGAGACCATCCTGGTGAGACTAGACTCCCAGGGCTCGGACGATCTGAACTA
TGTGCCCGGGGACCATCTCGCCGTCTTCCCGGCCAACGAGGACCACCTTGTGCAGGCCGTGC
TGGACCGACTGGACAACGCTCCCGACCCGGACAGTATCGTCAACATGGAGGTTCTCCAAGAG
AAGCAGACGCCGCTAGGAGCCATACGAACATGGATGACGAGCGAGAGACTGCCCCCCTGCTC
CCTCCGGACGGCCCTGTCTCGGTACTTTGACATCACGACGCCTCCATCGCCTCAGTTACTGC
AGCATCTGGCTACACAAGCTACAGATGAGGAGGAGAAGAAAGAACTGGAGGTTTTGGGAAAG
GGTGACGCGAGGTATGAAGACTGGAAGTTTGAGCGTACCCCGAACCTCGTAGAGGTCCTTGA
GGACTACCCGTCCCTGAAGGTGGCTCCGACCCTCCTGCTGAGTCAGCTACCCTTCCTGCAGC
AGCGGTACTACTCCATCAGCTCATCACAGAGGATGTACCCGGGAGAGATCCACGCTACGGTC
GCAGTGGTCAGGTTTGCCACTCAGGGTGGCGTGGGTCCTACCCACAACGGAGTCTGCTCATC
TTGGTTGAACCGAATCGAGAAGGATGATATCGTACCTTGTTTCGTGCGGGGGGCACAAAACT
TCCATCTGCCCGAGGACCCCACGGTTCCGCTGATGATGGTAGGTCCGGGGACTGGCATCGCG
CCGTTCCGCAGCTTCTGGCAGCACAGGCAGATGGAGGTCACCTCTGGTGACCCACATCATCG
GCCAAAGTACGGCCAGATGACGTTAGTATTCGGCTGTCGGCAGTCGAAAATGGACGACATCT
ACAAGCACGAGACAGCACAGGCAAAGGAGGACGGCGCCTTGACTGAGGTCTACACGGCTCTG
TCCAGGGAGCCGGGCGTACCAAAGTCGTATGTCCAGAACGTCATCTTGGACCTGATTCCCGA
GAAGGTGTGCGACCTCCTGATGAAGCATAACGGCCATTTCTACGTCTGCGGGGACGTTTCCA
TGGCAGCAGACGTCTGCAACACGCTGGAGAAGGCCATGGAAAAACAGCAGGGCATGACCCCC
AACAAGGCCAAGGACTTCGTCGACAAGCTGAAGGACTGCAACCGATACCATGAGGACATCTT
TGGGGTAACCTTGAGGACCCAGGAGGTGACTGACCGTGTGCGCTCGGCCGCGCGTAAGAACT
GGGTGCGGGTGAAGAGACTGCGGCCCAGCACGGTGGTCCCGCCCACCAGAGGTCTCTCACCG
GTACGTGAGAGCTCGTCCTTATCGTCTACGCCCATGTCCACGCCGTCCGCCACTCCCGTCAA
CTCTCCCTACTCCTCACCGCTGCATCGACCCGATCCGACCGTGTGTGCCAACGGCGATTTAC
CAAGTCTACACATTGAGGAATAA

Supplementary Table S4
NOS sequences accession number used in the phylogenetic study.

species	NOSI	NOSII	NOSIII
Homo sapiens	AAB49040	AAB60366	AAA36365

species	NOSA	NOSB	NOSC
Asymmetron lucayanum	GESY01069733<br>GESY01069734<br>GESY01069736<br>GESY01069735<br>GESY01069737<br>GESY01069738	GETC01128572<br>GETC01128573	GETC01013682<br>GETC01013681<br>GESY01015049<br>GESY01007340<br>GESY01007341<br>GETC01000005<br>GESY01098362<br>GETC01143078
Branchiostoma lanceolatum	Suppl. Figure 2	Suppl. Figure 2	Suppl. Figure 2
Branchiostoma floridae	XP_002608547	XP_019636970	XP_002605826
Branchiostoma belcheri	XP_019623080	XP_019636970	XP_019631555
Ciona intestinalis	XP_009861972		
Ciona savignyi	ENSCSAVG00000009725		
Strongylocentrotus purpuratus	XP_003729305		

**A. lucayanum NosC* gene coding sequence was very fragmented in short contigs. Two of these, GESY01098362 and GETC01143078, overlapped and we reconstructed a longer sequence, which was used for the phylogenetic analysis.

Supplmentary File S5

```
                                1
Homo_sapiens_nNOS               ETGCTEYICM GSIMHPQHAR RPEDVRTKGQ LFPLAKEFID QYYSSIKRFG
Homo_sapiens_iNOS               ILTCRSKSCL GSIMTPLTRG PRDKPTPPDE LLPQAIEFVN QYYGSFKEAK
Homo_sapiens_eNOS               DGPCTPRRCL GSLVFPLQGR PSPGPPAPEQ LLSQARDFIN QYYSSIKRSG
Branchiostoma_floridae_NOSA     VNPCSDGKCL GPLMRPRPFT TPGYFRPKEE VLEQAEKFIF EYYASLKRSD
Branchiostoma_floridae_NOSB     VSPCSSGKCM GSIVRQREPF PPYYKRPKEE VLEQAKEFID DYYASMKKEN
Branchiostoma_floridae_NOSC     VNPCSGTKCL GSLMRPKSGR PAGEVRPKEE VLEHAKEFLD EFFASIKRAN
Branchiostoma_belcheri_NOSA     LNPCSDGRCL GPLMRPRPYT APGYFRPKEE VLEQAKKFIF EYYASLKRSD
Branchiostoma_belcheri_NOSB     VSPCSSGKCM GSVVQQRQPF PPDYKRPKEE VLEQAKEFID DYYASLKKAN
Branchiostoma_belcheri_NOSC     MNPCSGTKCL GSLMRPKSTR PAGEVRPKEE VLEHAKEFLD EFFASIKRAN
Branchiostoma_lanceolatum_NOSA  LNPCNDGRCL GPLMRPRPFT APGYFRPKEE VLEQAKKFIF EYYASLKSSD
Branchiostoma_lanceolatum_NOSB  VAPCSSGKCM GSVVRQREPF PPGYKRPKEE VLEQAKEFID DYYASMKKEN
Branchiostoma_lanceolatum_NOSC  INPCSGTKCL GSLMRPKSAR PAGEPRPKDE VLEHAKEFLD EFFASIKRAN
Asymmetron_lucayanum_NOSA       VTPCSHGRCV GSLMRPRPYT APGYFRPKDE VLQQAKDFIF DYYASLKKSS
Asymmetron_lucayanum_NOSB       LNPCTSGKCM GSTVRQRQYF PPQYKRPNEE ILEQAKEFID DYYASMKKAN
Asymmetron_lucayanum_NOSC       -NPCSGTKCL GSLMRPQVKR PAGTARSKEE VLEHAKEFMD DFFASIKRAN
Ciona_intestinalis_NOS          INHCAPKKCN GSLMQPMVCD PRDGKPSVES VRKLAKDFID QYFASMKRND
Ciona_savignyi_NOS              ---------- ---------- ---------- ---------- ----KHSRND
Strongylocentrotus_purpuratus_NOSa ETSKRSGSAN NDSVT-SPTR QKPSMRSQDE KIRLAREFID QYYAHAQRSG

                               51
Homo_sapiens_nNOS               SKAHMERLEE VNKEIDTTST YQLKDTELIY GAKHAWRNAS RCVGRIQWSK
Homo_sapiens_iNOS               IEEHLARVEA VTKEIETTGT YQLTGDELIF ATKQAWRNAP RCIGRIQWSN
Homo_sapiens_eNOS               SQAHEQRLQE VEAEVAATGT YQLRESELVF GAKQAWRNAP RCVGRIQWGK
Branchiostoma_floridae_NOSA     AADRDRRWSD VQVQIEEKGV YHLTYDELAF AAKMSWRNSP RCIGRIQWSK
Branchiostoma_floridae_NOSB     SEDHERRWRD IQAQVAETGL YDLEYDELLY GAKMAWRNAP RCIGRYQWNN
Branchiostoma_floridae_NOSC     TQAHKQRWAE AKAQIEEKGW YELTQMELTY GAKLAWRNAP RCVGRIQWSK
Branchiostoma_belcheri_NOSA     VADRDRRWRD VQVQVEEKGV YHLTYEELVY GAKMSWRNAP RCIGRIQWSK
Branchiostoma_belcheri_NOSB     SAEHEKRWQD IQAQVSETGL YDLEYDELLY GAKMAWRNAP RCIGRYQWNN
Branchiostoma_belcheri_NOSC     TQAHKQRWAE AKSQIEEKGW YELTQMELTY GAKLGWRNAP RCVGRIQWSK
Branchiostoma_lanceolatum_NOSA  AGERDRRWRD VQVQVEEKGV YHLTYDELLY GAKMSWRNAP RCIGRIQWSK
Branchiostoma_lanceolatum_NOSB  SAEHERRWID IQAQVEEKGL YDLEYDELLY GAKMAWRNAS RCIGRYQWNN
Branchiostoma_lanceolatum_NOSC  TQAHKQRWAE AKVQIEEKGW YELTQMELTY GAKLAWRNAP RCVGRIQWSK
Asymmetron_lucayanum_NOSA       ADDRDRRWAD VQAQVEERGV YQLTYDELVY GAKMAWRNSP RCIGRIQWSK
Asymmetron_lucayanum_NOSB       STEHDRRWRD IQAQVEEKGV YDLTYDELLY GAKMAWRNAP RCIGRYQWNN
Asymmetron_lucayanum_NOSC       TRAHKQRWAE VEAQIEEKGL YELTQMELTY GAKLGWRNAP RCVGRIQWSK
Ciona_intestinalis_NOS          SEAHGARLDE VMNSIDSTGT YELTKNELIF GAKTAWRNAP RCIGRIQWNK
Ciona_savignyi_NOS              SEAHSSRLAE VLASISSTGT YELTKNELIF GAKTAWRNAP RCVGRIQWNK
Strongylocentrotus_purpuratus_NOSa TVSHERRLYE VQMSLKESGN YHLTEAELIF GAKSAWRNLP QSRCRIYWNK

                              101
Homo_sapiens_nNOS               LQVFDARDCT TAHGMFNYIC NHVKYATNKG NLRSAITIFP QRTDGKHDFR
Homo_sapiens_iNOS               LQVFDARSCS TAREMFEHIC RHVRYSTNNG NIRSAITVFP QRSDGKHDFR
Homo_sapiens_eNOS               LQVFDARDCR SAQEMFTYIC NHIKYATNRG NLRSAITVFP QRCPGRGDFR
Branchiostoma_floridae_NOSA     LQLFDAREVT TARGMFEAIC RHVKWCTNDG NIRSAITVFP PRTDGRHDYK
Branchiostoma_floridae_NOSB     LQVIDHRNVK TAKEMFDAVC DHVRYATKDG QMKTAISIFP MRTDGKHDYK
Branchiostoma_floridae_NOSC     LQVFDARYVT TARGMYEAIC NHIKYTTNKG NLRSAITIFP ARTDGKHDFK
Branchiostoma_belcheri_NOSA     LQLFDARDVT TARGMFEAIC RHIKWCTNGG NIRSAITVFP PRTDGRHDYK
Branchiostoma_belcheri_NOSB     LQVIDYRNVK TAKEMFDAVC DHVRYATKDG QMKTAISIFP MRTDGKHDWK
Branchiostoma_belcheri_NOSC     LQVFDARYVT TARGMYEAIC NHIKYATNKG NLRSAITIFP ARTDGKHDFK
Branchiostoma_lanceolatum_NOSA  LQLFDARDVT TARGMFEAIC RHIKWCTNGG NIRSAITVFP PRTDGRHDYK
Branchiostoma_lanceolatum_NOSB  LQVIDYRNVK TAQEMFDAAC DHIRYATKDG QMKTAISIFP MRTDGKHDYK
Branchiostoma_lanceolatum_NOSC  LQVFDARYVT TARGMYEAIC NHIKYATNKG NLRSAITIFP ARTDGKHDFK
Asymmetron_lucayanum_NOSA       LQVFDARDVT TARGMFEAIC RHIKWATNGG NLRSAITVFP PRTDGRHDYK
Asymmetron_lucayanum_NOSB       LQVLDQRHVK TAQEMFDAVC EHVRYATKDG QMKTAISIFP VRTDGKHDYK
Asymmetron_lucayanum_NOSC       LQVFDARYVT TARGMFEAIC NHIKYATNKG NIRSAITIFP ARTDGKHDFK
Ciona_intestinalis_NOS          LHVFDCRKVT STYEMFEAIC SHLKYATNKG NIRSCITFFP PRNDVKTEFR
Ciona_savignyi_NOS              LHVFDCRKVT STREMFDAIC VHLKYATNKG NIRSCITFFP PRNDVKTEFR
Strongylocentrotus_purpuratus_NOSa LQVFDARKVT NSRDMFEMLC KHLLYATNKG NVRPAITVFP ARNDNQTDFR
```

```
                                       151
Homo_sapiens_nNOS                      VWNSQLIRYA GYKQPDGSTL GDPANVQFTE ICIQQGWKPP RGRFDVLPLL
Homo_sapiens_iNOS                      VWNAQLIRYA GYQMPDGSIR GDPANVEFTQ LCIDLGWKPK YGRFDVVPLV
Homo_sapiens_eNOS                      IWNSQLVRYA GYRQQDGSVR GDPANVEITE LCIQHGWTPG NGRFDVLPLL
Branchiostoma_floridae_NOSA            VWNGQFLKYA AYQLPDGSIL GDPINLEFTE VCQALGWKGE GTRFDLLPLV
Branchiostoma_floridae_NOSB            FWNKYLFQYA AYQQPDGSVI GDPANLELTE VCHSLGWKGK GTPYDFLPAI
Branchiostoma_floridae_NOSC            VWNSQFVRYA GYKQPDGSIV GDPASVEFTE ICQSLGWKGK NGPFDVLPLV
Branchiostoma_belcheri_NOSA            VWNGQFMKYA AYQLPDGSIL GDPINLDFTE VCQALGWKGA GTRFDILPLV
Branchiostoma_belcheri_NOSB            FWNKLLFQYA GYEQPDGSVI GDPGSLELTK VCQSLGWKGK GTPYDFLPAI
Branchiostoma_belcheri_NOSC            VWNAQFIRYA GYKQPDGSVI GDPASVEFTE ICQSLGWKGK NGPFDVLPLV
Branchiostoma_lanceolatum_NOSA         VWNGQFLKYA AYQLPDGSIL GDPINLEFTE VCQALGWKGE GTRFDLLPMV
Branchiostoma_lanceolatum_NOSB         FWNKYLFQYA GYQQPDGSVI GDPANVELTE VCQSLGWKGK GTPYDFLPAI
Branchiostoma_lanceolatum_NOSC         VWNSQFVRYA GYKQPDGSVV GDPASVEFTE ICQSLGWKGK NGPFDVLPMV
Asymmetron_lucayanum_NOSA              VWNGQFMKYA AYEMPDGSIL GDPINLDFTE VCQALGWKGA GSGYDLLPLV
Asymmetron_lucayanum_NOSB              FWNNFLFRYA GYTQPDGSVI GDPDMVEFTE VCQSLGWRGK GTPYDYLPAI
Asymmetron_lucayanum_NOSC              IWNAQFIRYA GYKSX----- ---------- ------WKGK NGPFDVLPLL
Ciona_intestinalis_NOS                 VWNSQLIRYA CYRQEDGSLV GDPANLELTE LCERLGWKGK GGRFDLLPLL
Ciona_savignyi_NOS                     VWNSQLIRYA CYRQEDGSLV GDPANLELTE LCMRLGWKGK GGRFDLLPLL
Strongylocentrotus_purpuratus_NOSa     IWNSQLIGYA GYRQPDGSVL GDPSNLVFSE VCRTLGWRSD RTRFDVLPLV

                                       201
Homo_sapiens_nNOS                      LQANGNDPEL FQIPPELVLE VPIRHPKFEW FKDLGLKWYG LPAVSNMLLE
Homo_sapiens_iNOS                      LQANGRDPEL FEIPPDLVLE VAMEHPKYEW FRELELKWYA LPAVANMLLE
Homo_sapiens_eNOS                      LQAPDEPPEL FLLPPELVLE VPLEHPTLEW FAALGLRWYA LPAVSNMLLE
Branchiostoma_floridae_NOSA            LSANGEDPEW FDIPRDIVME VNITHPKYDW FEELGIKWYC VPAVSNMLFD
Branchiostoma_floridae_NOSB            VSANGEDPVI FDWPEDVPLE VELRHPKYDW FEELAIKANC IPMQGDMLFD
Branchiostoma_floridae_NOSC            LSANGQDPEL FELSKDLVLE VELKHPKYGW FKELGMKWYA LPAVANMLFD
Branchiostoma_belcheri_NOSA            LSCNGDDPEW FDIPKDIVME VNITHPKYDW FEELGIKWYC LPAVSNMLFD
Branchiostoma_belcheri_NOSB            VSANGEDPVV FDWPEDVPLE LELRHPKHDW FEELAIKVNC IPMQGDMLFD
Branchiostoma_belcheri_NOSC            LSANGQDPEI FELSKDLVLE VEFKHPKYDW FKELGIKWYA LPAVANMLFD
Branchiostoma_lanceolatum_NOSA         LSANGEDPEW FDLPKDIVME VNITHPKYDW FEELGIKWYC VPAVSNMLFD
Branchiostoma_lanceolatum_NOSB         VSANGEDPVI FEWPEDVPLE LELRHPKHDW FEELAIKVNC VPLQGDMLFD
Branchiostoma_lanceolatum_NOSC         LSANGQDPEL FELSRDLVLE VELKHPKYAW FKELGMKWYA LPAVANMLFD
Asymmetron_lucayanum_NOSA              LSANGEDPEW FDIPKDIVME VNITHPKYDW FSELGIKWYC VPAVSNMLFD
Asymmetron_lucayanum_NOSB              VSANGEDPVF FDWPEDVPLE LVLRHPKYDW FEELNIKVNC IPMQGDLLFD
Asymmetron_lucayanum_NOSC              LSANGQDPEL FELSKDLVLE VEIKHPKYEW FKELGIIWYA VPAVANMLFD
Ciona_intestinalis_NOS                 LQANGEDPEL YELPSNLVLE VKLRHPDQEW LEKMNLKWYA VPAVSNIMLN
Ciona_savignyi_NOS                     LQANGEDPEL YELPANLVLE VKLRHPEQDW LEQMNLKWYA VPAVSNILLD
Strongylocentrotus_purpuratus_NOSa     LQVNGEDPEV FEIPPELIHE VELAHPELRW FMDLKLKWYS MPSYSNFLLD

                                       251
Homo_sapiens_nNOS                      IGGLEFSACP FSGWYMGTEI GRDYCDNSRY NILEEVAKKM NLDMRKTSSL
Homo_sapiens_iNOS                      VGGLEFPGCP FNGWYMGTEI GRDFCDVQRY NILEEVGRRM GLETHKLASL
Homo_sapiens_eNOS                      IGGLEFPAAP FSGWYMSTEI GRNLCDPHRY NILEDVAVCM DLDTRTTSSL
Branchiostoma_floridae_NOSA            CGGLEFTAAP FSGWYMGTEV GRDLCDANRL NITEAVGKRM GLDVARNSSL
Branchiostoma_floridae_NOSB            VGGMQFPACP ITGWFQSTEP VRDLLDENRY NMTEIIAKKL GYDTSRYNSL
Branchiostoma_floridae_NOSC            VGGVEFPAAP FSGWYMCTEI GRDLCDINRY NFTEQIAKRM GLDTGRASSL
Branchiostoma_belcheri_NOSA            CGGLEFTAAP FSGWYMGTEV GRDLCDVNRL NITEEVGKRM GLDIARNSSL
Branchiostoma_belcheri_NOSB            VGGMQFPACP ITGWFQSTEP VRDLLDENRY NMTEMIAKKL GYDTSRYNSL
Branchiostoma_belcheri_NOSC            VGGVEFPAAP FSGWYMCTEI GRDLCDVNRY NFTEQIAKRM GLDTGRASSL
Branchiostoma_lanceolatum_NOSA         CGGLEFSAAP FSGWYMGTEV GRDLCDENRL NITEAVGKRM GLDVARNSSL
Branchiostoma_lanceolatum_NOSB         VGGMQFPACP ITGWFQSTEP VRDLLDENRY NMAEIIAKKL GYDTSRYNSL
Branchiostoma_lanceolatum_NOSC         VGGVEFPAAP FSGWYMCTEI GRDLCDINRY NYTEQIAKRM GLDTGRASSL
Asymmetron_lucayanum_NOSA              CGGVEFSAAP FSGWYMGTEV GRDLCDVNRL NITETVGRRM GLDIARNSSL
Asymmetron_lucayanum_NOSB              CGGMHFPACP ITGWFMSTEP VRDLLDENRY NFTEMIAKKL GYDTSRYNSL
Asymmetron_lucayanum_NOSC              VGGIEX---- ---------- ---------- ---------- ----------
Ciona_intestinalis_NOS                 IGGVEFPGVP FNGWFMGTEI GRDLLDTARY NLILPVAEAM GLNTKKTASL
Ciona_savignyi_NOS                     IGGVEFPAVP FNGWFMGTEI GRDLVDTSRY NMILPMAEAM GLNTKKTSSL
Strongylocentrotus_purpuratus_NOSa     VGGIQFPACP INEWYVASDI ANAMCAPKRY NMLTMIAERL GLDISCTDSL

                                       301
Homo_sapiens_nNOS                      WKDQALVEIN IAVLYSFQSD KVTIVDHHSA TESFIKHMEN EYRCRGGCPA
Homo_sapiens_iNOS                      WKDQAVVEIN IAVLHSFQKQ NVTIMDHHSA AESFMKYMQN EYRSRGGCPA
Homo_sapiens_eNOS                      WKDKAAVEIN VAVLHSYQLA KVTIVDHHAA TASFMKHLEN EQKARGGCPA
Branchiostoma_floridae_NOSA            WKDAVFVEVN IAVLHSFQRN NVTIMDHHTA SESFMKHLEN EQRLRGGCPS
Branchiostoma_floridae_NOSB            WKDAAFLECH IAVLHSFQMK GVMTQDPYTL TESFMKHMER EHRVRGGCPS
Branchiostoma_floridae_NOSC            WRDLALIEAN VAILHSFQTC NVTITDHHTA CESFMKHMEN EQRLRGGCPA
Branchiostoma_belcheri_NOSA            WKDAVLVEVN IAVLHSFQRS NATIMDHHTA SESFMKHLEN EQRLRGGCPS
Branchiostoma_belcheri_NOSB            WKDAAFLEVH IAVLHTFQMN GVMTQDPHTL TESFMKHMER EHRLRGGCPS
Branchiostoma_belcheri_NOSC            WRDLALIEAN VAILHSFQTG NVTITDHHTA CESFMKHLEN EQRLRGGCPA
Branchiostoma_lanceolatum_NOSA         WKDAVFVEVN IAVLHSFQRN NATIMDHHTA SETFMKHLEN EQRLRGGCPS
Branchiostoma_lanceolatum_NOSB         WKDAAFLEVH IAVIHSFQMK GVMTQDPYTL TESFMKHMER EHRVRGGCPS
Branchiostoma_lanceolatum_NOSC         WRDLAMIEAN VAILHSFQTG NVTITDHHTA CESFMKHMEN EQRLRGGCPA
Asymmetron_lucayanum_NOSA              WKDAVLVEVN IAVLHSFQSN NVTISDHHTA SETFIKHLEN EQRLRGGCPS
Asymmetron_lucayanum_NOSB              WKDAAFLEVH LAVLHSFQLE GVMTQDPHTL TESFMKHMEK EHRLRGGCPS
Asymmetron_lucayanum_NOSC              ---------- ---------- ---------- ---------- ----------
Ciona_intestinalis_NOS                 WKDKAILELN IAVLHSFQDD NVTIMDHHVA TESFMKHFSN EMKLRGGCPG
Ciona_savignyi_NOS                     WKDKAILELN LAILHSFQSD NVTIMDHHVA TESFMKHFAN EVKLRGGCPG
Strongylocentrotus_purpuratus_NOSa     WRDRTLVEMN VAVLHSFQQY GVTIMDHHTS SAEFMKFFAS EHEIRQGCPT
```

```
                                    351
Homo_sapiens_nNOS                   DWVWIVPPMS GSITPVFHQE MLNYRLTPSF EYQPDPWNTH VWKGTNG-TK
Homo_sapiens_iNOS                   DWIWLVPPMS GSITPVFHQE MLNYVLSPFY YYQVEAWKTH VWQDEKR--K
Homo_sapiens_eNOS                   DWAWIVPPIS GSLTPVFHQE MVNYFLSPAF RYQPDPWKGS AAKG---TGI
Branchiostoma_floridae_NOSA         DWVWIVPPMS ASLTEVFHQE MANYHVRPSF EYQEDAWLVH LWRKKPKENP
Branchiostoma_floridae_NOSB         DWVWIVPPSC SSLTPVFHQE MLNYHLRPAL EYQDAGWKIH TWKTPPTIRT
Branchiostoma_floridae_NOSC         DWVWIVPPIS GSLTPVFHQE MISYYLRPSY EYQEDAWMTH VWKKKEDGKA
Branchiostoma_belcheri_NOSA         DWVWIVPPMS ASLTEVFHQE MANYHVRPSF EYQEDAWLVH LWRKKPKENP
Branchiostoma_belcheri_NOSB         DWVWIVPPSC SSLTPVFHQE MLNYHLRPAL EYQAAGWTIH TWKTPPTIRT
Branchiostoma_belcheri_NOSC         DWVWIVPPVS ASLTPVFHQE MISYYLRPSY EYQEDAWKTH VWKKKEDGKT
Branchiostoma_lanceolatum_NOSA      DWVWIVPPMS ASLTEVFHQE MANYHVRPSF EYQEDAWLLH LWRKKPEENP
Branchiostoma_lanceolatum_NOSB      DWVWIVPPSC SSLTPVFHQE MLNYHLRPAL EYQEAGWKIH TWKTPPTIRT
Branchiostoma_lanceolatum_NOSC      DWVWIVPPVS ASLTPVFHQE MISYYLRPSY EYQEDAWKTH VWKKKEDGKA
Asymmetron_lucayanum_NOSA           DWVWIVPPMS ASLTEVFHQE MAYYLVRPSF EYQDDAWLVH LWRKKPQENP
Asymmetron_lucayanum_NOSB           DWVWITPPSC SSLTPVFHQE MLNYHLRPAL EYQEAGWKIH EWKTLPTVRT
Asymmetron_lucayanum_NOSC           ---------- ---------- ---------- ----DAWKTH VWKKKEDGKA
Ciona_intestinalis_NOS              DWVWLVPPVS GSATPIFHQE ILNYTLNPAC LYQPDPWHYY KWKDSVMKKR
Ciona_savignyi_NOS                  DWVWLVPPVS GSATPVFHQE ILNYTLNPSC LYQPDPWQYY KWKDTVMVKR
Strongylocentrotus_purpuratus_NOSa  DWRAVVSPLT ASLTSLFHQE TLNYQLMPTF TVQDNPWEVP EWKSKLSTDR

                                    401
Homo_sapiens_nNOS                   RRAIGFKKLA EAVKFSAKLM GQAMAKRVKA TILYATETGK SQAYAKTLCE
Homo_sapiens_iNOS                   RREIPLKVLV KAVLFACMLM RKTMASRVRV TILFATETGK SEALAWDLGA
Homo_sapiens_eNOS                   TRKKTFKEVA NAVKISASLM GTVMAKRVKA TILYGSETGR AQSYAQQLGR
Branchiostoma_floridae_NOSA         PRRCSLKEVA LAVTFAAVLM KKALDKRVKT TILYATETGK SESFASSTLG
Branchiostoma_floridae_NOSB         KKRCSFKAAA MAVMFAAGLM RKTLNKRVKS TILFASQTGK AETFSHSLCD
Branchiostoma_floridae_NOSC         KRKFGFKEVA KAVKFSAKLM GKALAKRVKA TILYATETGK SERYAKTICE
Branchiostoma_belcheri_NOSA         KRKYRLKEVA LAVKFAAVLM KKALHKRVKT TILYATETGK SESFATSTLG
Branchiostoma_belcheri_NOSB         KERCSFKAAA MAVMFAAGLM RKTLNRRVKS TILFASQTGK AETFSHSLCD
Branchiostoma_belcheri_NOSC         KRKFGFKEVA KAVKFSAKLM GKALAKRVKA TILYATETGK SERYAKTVCE
Branchiostoma_lanceolatum_NOSA      KRKYRLKEVA LAVKFAAVLM KKALNKRVKT TILYATETGK SESFASSTLT
Branchiostoma_lanceolatum_NOSB      KKRCSFKAAA MAVMFASGLM RKTLNKRVKS TILFASQTGK AETFSNSLCD
Branchiostoma_lanceolatum_NOSC      KRKFGFKEVA KAVKFSAKLM GKALAKRVKA TILYATETGK SERYAKTVCE
Asymmetron_lucayanum_NOSA           TRKYRFKEVA LAVKFAAVLM KKALDKRVKA TILYATETGK SESFASSTLG
Asymmetron_lucayanum_NOSB           KERCSFKAAA LAVLFAAGSM RKALDKRVKT TILFASQTGK AETFSHSVCD
Asymmetron_lucayanum_NOSC           KRKFGFKEVA KAVKFSAKLM GKALAKRVKA TILFATETGK SERYAKTVCE
Ciona_intestinalis_NOS              KNKLHFKELA RSVLMTSRMM TKAMANRIKA TILYASETGK SYAYSKSLQE
Ciona_savignyi_NOS                  KNKLHFKELA KSVMVTSRMM KKAMANRIKT TILYATETGK SYAYSLILQE
Strongylocentrotus_purpuratus_NOSa  LRSPG----A DPSRFSSRIL RRALSGTFKA TVLYASEFGK SEYYAKKVYE

                                    451
Homo_sapiens_nNOS                   IFKHAFDAKV MSMEEYDIVH LEHETLVLVV TSTFGNGDPP ENGEKFGCAL
Homo_sapiens_iNOS                   LFSCAFNPKV VCMDKYRLSC LEEERLLLVV TSTFGNGDCP GNGEKLKKSL
Homo_sapiens_eNOS                   LFRKAFDPRV LCMDEYDVVS LEHETLVLVV TSTFGNGDPP ENGESFAAAL
Branchiostoma_floridae_NOSA         IFKHAFDAKM MCMDEYDITH LEKEELVIVV TSTFGNGDPP DNGEAFGQAL
Branchiostoma_floridae_NOSB         LYKHAFDAKV ICMDEYDVTQ MDKEQLVVIV TSTFGNGDPP DNGETFARAL
Branchiostoma_floridae_NOSC         IFKHAFDAKV MCMDDYDIMH LEHETLVIVV TSTFGNGDPP DNGESFGQTL
Branchiostoma_belcheri_NOSA         IFKHAFDAKI MCMDEYDITN LEKEELVIVV TSTFGNGDPP DNGEAFGQAL
Branchiostoma_belcheri_NOSB         LYKHAFHAKV VCMDEYDMTQ LDKEQLVIVV TSTFGNGDPP DNGKTFARAL
Branchiostoma_belcheri_NOSC         IFKHAFDAKV MCMDDYDIMH LEHETLVIVV TSTFGNGDPP DNGESFGQAL
Branchiostoma_lanceolatum_NOSA      IFKHAFDAKM MCMDEYDITN LEKEELVIVV TSTFGNGDPP DNGEAFGQAL
Branchiostoma_lanceolatum_NOSB      LYKHAFDAKV VCMDEYDMAQ LDKEQLVVIV TSTFGNGDPP DNGETFARAL
Branchiostoma_lanceolatum_NOSC      IFKHAFDAKV MCMDDYDIMH LEHETLVIVV TSTFGNGDPP DNGESFGQAL
Asymmetron_lucayanum_NOSA           IFKHAFDAKM MCMDDYDVTE LEKEDLVIIV TSTFGNGDPP DNGEAFGQAL
Asymmetron_lucayanum_NOSB           LYKHAFDAKV VCMDEYDISR LSKEQLVVVV TSTFGNGDPP DNGETFARAL
Asymmetron_lucayanum_NOSC           IFKHAFDAKV MCMDDYDIMH LEHETLVIVV TSTFGNGDPP DNGESFGQAL
Ciona_intestinalis_NOS              IFNHAFDAKV VPMDQYDICN LEYEALVLIV TSTFGNGDPP ENGEYLAQAL
Ciona_savignyi_NOS                  IFNHAFDAKV VAMDQYDICN LEYESMLLVV TSTFGNGDPP ENGEDF--LL
Strongylocentrotus_purpuratus_NOSa  TFRKAFDIRL LRMDEYDVMN LEHENLLLVV TSTLGNGEPP SNGQEFCRYL

                                    501
Homo_sapiens_nNOS                   MEMRHPNSVQ EERKSVRFSV FGLGSRAYPH FCAFGHAVDT LLEELGGERI
Homo_sapiens_iNOS                   FMLKELNNK- -----FRYAV FGLGSSMYPR FCAFAHDIDQ KLSHLGASQL
Homo_sapiens_eNOS                   MEMSGPYNSS PRPEQLRFCV FGLGSRAYPH FCAFARAVDT RLEELGGERL
Branchiostoma_floridae_NOSA         LHMRHPPGSD GKKRSVRYGV FALGSRAYPH FCAFGHAIDT LFEELGAERI
Branchiostoma_floridae_NOSB         MQMKKQNGQT SPGDSVRYAV FALGSRAYPH FCGFGHSLNT LLEQLGAQRV
Branchiostoma_floridae_NOSC         LEMRHPPMDN NDNRPVRFSV FGLGSRAYPG FCAFAHAVDT LFGELGGERI
Branchiostoma_belcheri_NOSA         LHMRHPPGND GKKKSVRYCV FALGSRAYPH FCAFGHAIDT LFEELGAERI
Branchiostoma_belcheri_NOSB         MQMKNQKDQT SPDNNVRFAV FALGSRAYPH FCGFGHSVDT LLDQLGAQRI
Branchiostoma_belcheri_NOSC         LEMRHPPMDN NDNRPVRFSA FGLGSRAYPG FCAFAHAVDT LLGELGGERI
Branchiostoma_lanceolatum_NOSA      LHMRHPPGSD KKRSGVRYGV FALGSRAYPH FCAFGHAIDT LFESLGAERI
Branchiostoma_lanceolatum_NOSB      MQMKNQNGQA SPRDSVRFAV FALGSRAYPH FCAFGHSVDT LLEQLGARRV
Branchiostoma_lanceolatum_NOSC      LEMRHPPMDN NDNRPVRFSA FGLGSRAYPG FCAFAHAVDT LFGELGGERI
Asymmetron_lucayanum_NOSA           LHMRHPPGSV GKKKSVRYAV FALGSRAYPH FCAFGHAINT LFEELGAERI
Asymmetron_lucayanum_NOSB           MQMKNKSGQ- -----VRFAV FALGSRAYPH FCGFGHSVDT LFEQLGARRV
Asymmetron_lucayanum_NOSC           LEMRHPPTDN NDNKA----- ---------- ---------- ----------
Ciona_intestinalis_NOS              QKIVRDSKED QKKAEVKFAV FGLGSRAYPH FCAFAHAMDT FLGELEGERL
Ciona_savignyi_NOS                  FSFINIEHGI HKIKSSRFAV FGLGSRAYPH FCAFAHAVDT LLGELEGERV
Strongylocentrotus_purpuratus_NOSa  MEMSDNNNNR SPVPQLSYGV FGLGSSIYPY YCAFARGVDQ MLDSLGGDRL
```

```
                                   551
Homo_sapiens_nNOS                  LKMREGDELC GQEEAFRTWA KKVFKAACDV FCVGDDVNIE KANNSLISNS
Homo_sapiens_iNOS                  TPMGEGDELS GQEDAFRSWA VQIFKAACET FDVRGKQHIQ IP--KLYTST
Homo_sapiens_eNOS                  LQLGQGDELC GQEEAFRGWA QAAFQAACET FCVGEDA--K AAARDIFSPS
Branchiostoma_floridae_NOSA        HPVGEGDELC GQEESFRAWA KGAFKSACER YDVGHDVNME EANASLLGSS
Branchiostoma_floridae_NOSB        HAVGEGDELC GQEESFRAWA ESAFKSACLS YDVGHGVDMS EATANLLGSS
Branchiostoma_floridae_NOSC        YKMGEGDELC GQEESFRKWA KGVFKAACDT FCVGDELNMS EASATLLNST
Branchiostoma_belcheri_NOSA        HPVGEGDELC GQEESFRAWV KGAFKSACER YDVGHDVNME EANASLLGSS
Branchiostoma_belcheri_NOSB        HAVGEGDELC GQEESFRTWA ESAFKSACLS YDVGHGVDMN EATANLLGSS
Branchiostoma_belcheri_NOSC        YKMGEGDELC GQEESFRKWA KGVFRAACDT FCVGDDLNMN EASATLLNTT
Branchiostoma_lanceolatum_NOSA     HPVGEGDELC GQEESFRVWV KGAFKSACER YDVGHYVNME EANASLLGSS
Branchiostoma_lanceolatum_NOSB     HAVGEGDELC GQEESFRAWA ESAFKSACLS YDVGHGVDMN EATANLLGSS
Branchiostoma_lanceolatum_NOSC     YKMGEGDELC GQEESFRKWA KGVFKAACDT FCVGDDLNMS EASSTLLNST
Asymmetron_lucayanum_NOSA          HPVGEGDELC GQEESFRAWV KGAFKSACER FDVGHDVNME EANASLLGNS
Asymmetron_lucayanum_NOSB          HTVGEGDELC GQEESFRAWA ESAFKSSCLS FDVGHDVDMN EATANLLGSS
Asymmetron_lucayanum_NOSC          ---------P GHTKRLSSIS SSAT------ ---------- ----------
Ciona_intestinalis_NOS             MEIGEGDELC GQEESFRRWA QEVFTSACDT FCLREQVNLE DANASLSTSK
Ciona_savignyi_NOS                 KEIGEGDEMC GQEECFRNWA QQVFQGGGNS FDRLLIFNLE DANASLKSTK
Strongylocentrotus_purpuratus_NOSa VGMGWGDEMG GLDETFDAWL TKTFQKYAWL FFIFDYTVLS IFVCELILKG

                                   601
Homo_sapiens_nNOS                  WKRNKFRLTF VAEAPELTQG LSNVHKKRVS AARLLSRQNL QSPKSSRSTI
Homo_sapiens_iNOS                  WDPHHYRL-- VQDSQPLSKA LSSMHAKNVF TMRLKSRQNL QSPTSSRATI
Homo_sapiens_eNOS                  WKRQRYRLAQ AEGLQ-LLPG LIHVHRRKMF QATIRSVENL QSSKSTRATI
Branchiostoma_floridae_NOSA        WAPGKFRIVQ TKGLPEVLEG LSKLHRRTLV SSTVISRTQL QAPESGRQTC
Branchiostoma_floridae_NOSB        WAPDKFRLVQ AKSNRDLLEG LSTVHCKSVV SCSFISRTQL QAPDSSRQTI
Branchiostoma_floridae_NOSC        WAADKFRLIP AEGVKEIWEG LSKVHSRNVV PCRLISRENL QAPDSGRETI
Branchiostoma_belcheri_NOSA        WAPGKFRIVQ TKGLPEILEG LSKLHRRTVV SSTVISRTQL QAPESSRQTC
Branchiostoma_belcheri_NOSB        WAPDKFRLVQ AKSNKDLLEG LSTVHRKNVV PCTFISRTQL QAPDSSRQTI
Branchiostoma_belcheri_NOSC        WSADKFRLIP AEGVMEIWEG LSKVHSRTVV PCRLISRENL QAPDSGRETI
Branchiostoma_lanceolatum_NOSA     WAPGKFRVLQ TKGLPEILEG LSKLHRRTVV SSTVISRTQL QAPESSRQTC
Branchiostoma_lanceolatum_NOSB     WAPDKFRLVQ AKTKTDILQG LSTVHRKNIV PCTFISRTQL QAPDSSRQTI
Branchiostoma_lanceolatum_NOSC     WSPDKFRLIP AEGVKEIWEG LSKVHHRNVV PCRLISRENL QAPDSGRETI
Asymmetron_lucayanum_NOSA          WAPGKFHVVQ TKGLPEILEG LSKLHRRTVV SSTVISRTQL QAPESSRQTC
Asymmetron_lucayanum_NOSB          WAPDKFRLAQ AKGTRDLLEG LSTLHRKKVV SCTFISRTQL QAPDSSRQTI
Asymmetron_lucayanum_NOSC          ---------- ---------- ----ERRKKF SRQXISRTQL QAPESSRQTC
Ciona_intestinalis_NOS             FERTDFRVVH DKQVQPVIKT LGTMHRKKLS SCRLIRRKNL QSEESGRSTI
Ciona_savignyi_NOS                 FDKSNFRVVS DVQVEPV--- ---------- ---------- ---ESKRSTI
Strongylocentrotus_purpuratus_NOSa WNILDFFIIV ILLLGPKFLG SSRILRILRV LRAFRSLR-- -----SRSTI

                                   651
Homo_sapiens_nNOS                  FVRLHTNGSQ ELQYQPGDHL GVFPGNHEDL VNALIERLED APPVNQMVKV
Homo_sapiens_iNOS                  LVELSCEDGQ GLNYLPGEHL GVCPGNQPAL VQGILERVVD GPTPHQTVRL
Homo_sapiens_eNOS                  LVRLDTGGQE GLQYQPGDHI GVCPPNRPGL VEALLSRVED PPAPTEPVAV
Branchiostoma_floridae_NOSA        LVELETHGAQ ELKYVPGDHV AIFPANEDRL VQAILDRLEK GTNPDAVILI
Branchiostoma_floridae_NOSB        LVQLDTKGNK ELQFTPGDHL GVFPANEDRL VQEILDRIEG GTNPDDVLEI
Branchiostoma_floridae_NOSC        LVRLDSQGSD DLNYVPGDHL AVYPANEDHL VQAVLDRLDN APDPDCIVNM
Branchiostoma_belcheri_NOSA        LVQLETHGAH ELKYVPGDHV AVFPANEDRL VQAILDRLEK GTNPDAVIQI
Branchiostoma_belcheri_NOSB        LVQLDTKGRE ELKFSPGDHL GIFPANENHL VQRILGRIDG GTNPDDVLEI
Branchiostoma_belcheri_NOSC        LVRLDSQGSD DLNYVPGDHL AVFPANEDHL VQAVLDRLDN APDPDSIVNM
Branchiostoma_lanceolatum_NOSA     LVQLETHGAQ ELRYVPGDHV AVFPANEDRL VQAILDRVEK GTNPDAVIQI
Branchiostoma_lanceolatum_NOSB     LVRLDTKDNE ELEFTPGDHL GVFPANEDHL VQAILDRIEG GTKPDDVLEI
Branchiostoma_lanceolatum_NOSC     LVRLDSQGSD DLNYVPGDHL AVFPANEDHL VQAVLDRLDN APDPDSIVNM
Asymmetron_lucayanum_NOSA          LVRLETHGAH ELRYVPGDHV GIFPANEERL VREILDRLEQ GTNPDCVIQI
Asymmetron_lucayanum_NOSB          LVRLDTKGNE ELKFTPGDHL GVFPANEDHL VQAILDRVEG GTDPDEVLEV
Asymmetron_lucayanum_NOSC          LVRLGTHGDH ELRYASGDHV GIFPANEDRL VQEILDRMDK GTNPDDVIQT
Ciona_intestinalis_NOS             LIQLETKNCE NLNFCPGDHL GIYPRNDPDL VKELLSKIRD ATDENTVVVL
Ciona_savignyi_NOS                 LIQLEKKNSD VLNFQPGDHL GIYPRNDPAL VKALISKIRD ATDDSTVVRL
Strongylocentrotus_purpuratus_NOSa LVRLNTNKER GMAYQPGDHV AFFPSNENAL VARVLKRLDY QKSPDAIFKL

                                   701
Homo_sapiens_nNOS                  ELLERNTNWT DELRLPPCTI FQAFKYYLDI TTPPTPLQLQ QFASLATSEK
Homo_sapiens_iNOS                  EALESGSYWV SDKRLPPCSL SQALTYFLDI TTPPTQLLLQ KLAQVATEEP
Homo_sapiens_eNOS                  EQLEKGSGWV RDPRLPPCTL RQALTFFLDI TSPPSPQLLR LLSTLAEEPR
Branchiostoma_floridae_NOSA        EALEKKSTWT SHNRLPPCSL RTALSRYLDV TTPPSPQLLL YLAMHTTSSR
Branchiostoma_floridae_NOSB        EALKKLTTWM PFERLPPCSM RTALSRYLDV TTPPSPQLLG SLAVHTGEPG
Branchiostoma_floridae_NOSC        EVLEKQTSWM TSERLPPCSL RTALSRYFDI TTPPSPQLLQ HLATQATDEE
Branchiostoma_belcheri_NOSA        EALEKKSTWT PHDRLPPCSL RTALSRYLDV TTPPSPQLLL YLAMHATSNR
Branchiostoma_belcheri_NOSB        EALKKTVTWM PFERLPPCSL RTALSRYLDI TTPPSPQLLG SLAMHTNDTA
Branchiostoma_belcheri_NOSC        EVLEKQTTWM TSERLPPCSL RTALSRYFDI TTPPAPQLLQ HLATQATDEE
Branchiostoma_lanceolatum_NOSA     EALEKKISWT PHDRLPTCSL RTALSRYLDV TTPPSPQLLL YLAMHATSSR
Branchiostoma_lanceolatum_NOSB     EALKKLTTWM PFERLPPCSL RTALSRYLDI TTPPSPQLLG SLSMHTSAPS
Branchiostoma_lanceolatum_NOSC     EVLEKQTTWM TSERLPPCSL RTALSRYFDI TTPPSPQLLQ HLATQATDEE
Asymmetron_lucayanum_NOSA          EAQEKKSTWT THERLPPCSL RTALSRYLDI TTPPSPQLLL YLAMHATSSR
Asymmetron_lucayanum_NOSB          EALKKLTTWM PFERLPPCSL RTALSRYLDI TTPPTPQLLG SLSMHAAAPE
Asymmetron_lucayanum_NOSC          E--------- ---------- ---------- ---------- ----------
Ciona_intestinalis_NOS             EAKEKRSYWE EDTRLPPCTL NMAFSRYLDI TTPPTPQLLK LFSECDVNQE
Ciona_savignyi_NOS                 E------YWD ADTRLPSCTL TMAFSRFLDI TTPPTPQLLK LFSECNVNEE
Strongylocentrotus_purpuratus_NOSa EKLPRETVWT TVSRLPTCSL RTALERYLDL TTPPTPDFLR LLATRATDPR
```

```
                                     751
Homo_sapiens_nNOS                    EKQRLLVLSK GLQEYEEWKW GKNPTIVEVL EEFPSIQMPA TLLLTQLSLL
Homo_sapiens_iNOS                    ERQRLEALCQ -PSEYSKWKF TNSPTFLEVL EEFPSLRVSA GFLLSQLPIL
Homo_sapiens_eNOS                    EQQELEALSQ DPRRYEEWKW FRCPTLLEVL EQFPSVALPA PLLLTQLPLL
Branchiostoma_floridae_NOSA          ERAELEALGK GGARYEDWKF EAAPTLPEVL QDYPSLQVPP ALLLSQLPVL
Branchiostoma_floridae_NOSB          ERAELEELAK GGTKYENWKF ERAPTLPEVL QDYPSLQVPA ALLLSQLPVL
Branchiostoma_floridae_NOSC          EKKELEVLGK GDSRYEDWKF ERTPNLVEVL EDYPSLKVAP TLLLSQLPFL
Branchiostoma_belcheri_NOSA          ERAELQALGK GGLKYEDWKF EVAPTLPEVL QDYPSLQVPP ALLLSQLPVL
Branchiostoma_belcheri_NOSB          ERAGLQELAK GGSKYEDWKF EHAPTLPEVL QEFPSLTVPP ALLLSQLPVL
Branchiostoma_belcheri_NOSC          EKKELEVLGK GDSRYEDWKF ERTPNLVEVL EDYPSLKVAP TLLLSQLSFL
Branchiostoma_lanceolatum_NOSA       ERAELEALGK GGLRYEDWKF EAAPTLPEVL QHYPSLQVPP ALLLSQLPVL
Branchiostoma_lanceolatum_NOSB       ERAELEELAK GGTKYENWKF ERAPTLPEVL QEYPSLQVPP ALLLSQLPVL
Branchiostoma_lanceolatum_NOSC       EKKELEVLGK GDARYEDWKF ERTPNLVEVL EDYPSLKVAP TLLLSQLPFL
Asymmetron_lucayanum_NOSA            ERTELEALGK GGPRYEDWKF EVAPTLLEVL QDFPSLQVPP ALLLSQLPVL
Asymmetron_lucayanum_NOSB            EMAELEELGK GGKKYESWKF ERAPTLPEVL QNYPSLKVPP ALLLSQLPVL
Asymmetron_lucayanum_NOSC            ---------- ---------- ---------- ---------- ----------
Ciona_intestinalis_NOS               ESKRLEVLSQ GQSEYEDWKF LNNPNLIEVL NQFPSIQLEA ALLLKQLPLL
Ciona_savignyi_NOS                   QSTALRILGQ GESEYEDWKF SNNPNLVEVL NEFPSTQVDA SLLLKQLPLL
Strongylocentrotus_purpuratus_NOSa   DKEKLSILGA GCDEYEEWKW ENWPNMAEVL EGYPSLKVPA SLLLTQLPLL

                                     801
Homo_sapiens_nNOS                    QPRYYSISSS PDMYPDEVHL TVAIVSYRTR DGEGPIHHGV CSSWLNRIQA
Homo_sapiens_iNOS                    KPRFYSISSS RDHTPTEIHL TVAVVTYHTG DGQGPLHHGV CSTWLNSLKP
Homo_sapiens_eNOS                    QPRYYSVSSA PSTHPGEIHL TVAVLAYRTQ DGLGPLHYGV CSTWLSQLKP
Branchiostoma_floridae_NOSA          QQRYYSISSS PHMYPGQIHA TVDMVKYRTR GGKGPEHSGV CSNWLNTINI
Branchiostoma_floridae_NOSB          QQRYYSISSS PHMYRGEIHA TLAVVSYRSQ GGKGPIHNGV CSSWFNRIEE
Branchiostoma_floridae_NOSC          QQRYYSISSS QLMYPGEIHA TVAVVKFATQ GGVGPIHNGV CSSWLNRIEK
Branchiostoma_belcheri_NOSA          QQRYYSISSS PHMYPGQIHA TVAVVKYRTR GGKGPEHNGV CSNWLNNVKT
Branchiostoma_belcheri_NOSB          QQALFHTAPP PHMYPGQIHA TLAVVSYRTQ GGKGPTHNGV CSSWLNRIEE
Branchiostoma_belcheri_NOSC          QQRYYSISSS QRMFPGEIHA TVAVVRFATQ GGVGPIHNGV CSSWLNRIVK
Branchiostoma_lanceolatum_NOSA       QQRYYSISSS PHMYPGQIHA TVAVVKYRTR GGQGPEHSGV CSNWLNTIKS
Branchiostoma_lanceolatum_NOSB       QQRYYSISSS PHMYPGEIHA TLAVVSYRSQ GGKGPVHNGV CSSWFNRLEE
Branchiostoma_lanceolatum_NOSC       QQRYYSISSS QRMYPGEIHA TVAVVRFATQ GGVGPTHNGV CSSWLNRIEK
Asymmetron_lucayanum_NOSA            QQRYYSISSS PHMHPGEIHA TVAVVKYRTR GGQGPEHRGV CSNWLNTVKS
Asymmetron_lucayanum_NOSB            QQRYYSISSS PHMYPGEIHA TLAVVSFRAQ GGKGPVHNGV CSSWFNRIGD
Asymmetron_lucayanum_NOSC            ---------- ---------- ---------- ---------- ----------
Ciona_intestinalis_NOS               QARYYSISSS PEIFPGEIHC TVSVVTYHTR GGTGPLHHGV CSSYLNSITD
Ciona_savignyi_NOS                   QARYYSISSS PDVYPGEIHC TVSVVTYHTR GGDGPLHHGV CSSYLNSITD
Strongylocentrotus_purpuratus_NOSa   RPRYFSISSS PKQYPNEIHA TVAVVSYHPN GGRGPVHYGA CSSWLYRAWK

                                     851
Homo_sapiens_nNOS                    DELVPCFVRG APSFHLPRNP QVPCILVGPG TGIAPFRSFW QQRQFDIQHK
Homo_sapiens_iNOS                    QDPVPCFVRN ASAFHLPEDP SHPCILIGPG TGIAPFRSFW QQRLHDSQHK
Homo_sapiens_eNOS                    GDPVPCFIRG APSFRLPPDP SLPCILVGPG TGIAPFRGFW QERLHDIESK
Branchiostoma_floridae_NOSA          NESVPCFIRT AKNFHLPEDP SLPVLMVGPG TGIAPLRSFW QQRQVDIKTG
Branchiostoma_floridae_NOSB          GETVPCFVRP AKNFHLPDDP SMPVLMVGPG TGIAPLRSFW QQRQMEIKSG
Branchiostoma_floridae_NOSC          DDIVPCFVRA AQSFHLPEDP TVPLMMVGPG TGIAPFRSFW QHRQMEVTSG
Branchiostoma_belcheri_NOSA          NESVPCFIRT AKNFHLPEDS SLPVLMVGPG TGIAPLRSFW QQRQVDIKAG
Branchiostoma_belcheri_NOSB          GETVPCFVRT AKNFHLPDDP SVPVLMVGPG TGIAPLRSFW QQRQADIKSG
Branchiostoma_belcheri_NOSC          DDIVPCFVRA AQNFHLPEDP TVPLMMVGPG TGIAPFRSFW QQRQMEVGSG
Branchiostoma_lanceolatum_NOSA       NESVPCFIRT AKNFHLPENS SLPVLMVGPG TGIAPLRSFW QQRQVDIKAG
Branchiostoma_lanceolatum_NOSB       GETVPCFVRA AKNFHLPADP SMPVLMVGPG TGIAPLRSFW QQRQMDIKSG
Branchiostoma_lanceolatum_NOSC       DDIVPCFVRG AQNFHLPEDP TVPLMMVGPG TGIAPFRSFW QHRQMEVTSG
Asymmetron_lucayanum_NOSA            GDSVPSFIRT AKNFHLPENP SLPVLMVGPG TGIAPLRSFW QQRQVDIKSG
Asymmetron_lucayanum_NOSB            GETVPCFVRP AKNFHLPDDP TMPVLMVGPG TGIAPLRGFW QQRQMDISSG
Asymmetron_lucayanum_NOSC            ---------- ---------- ---------- ---------- ----------
Ciona_intestinalis_NOS               DEEVACFVRT APTFHLPLKD NIPCILVGPG TGIAPFRSFW QQRLAEIRKG
Ciona_savignyi_NOS                   EEEVPCFVRM APTFHLPTKE DAPCILVGPG TGIAPFRSFW QHRISQIQKG
Strongylocentrotus_purpuratus_NOSa   DEVIPAFVSS APYFHLPADT SVPVIMVGPG SGVAPFRSFW QDRQTDLRQG

                                     901
Homo_sapiens_nNOS                    GMCPMVLVFG CRQSKIDHIY REETLQAKNK GVFRELYTAY SREPDKPKKY
Homo_sapiens_iNOS                    GVGRMTLVFG CRRPDEDHIY QEEMLEMAQK GVLHAVHTAY SRLPGKPKVY
Homo_sapiens_eNOS                    GLTPMTLVFG CRCSQLDHLY RDEVQNAQQR GVFGRVLTAF SREPDNPKTY
Branchiostoma_floridae_NOSA          TTGDMTLVFG CRQSRLDHIY KEETAQARRD GALKDIHTAL SREPGTTKTY
Branchiostoma_floridae_NOSB          NHGDMTLVFG CRQSQIDHIY KEETAQAKSD GALTDIHTAY SREPGTPKTY
Branchiostoma_floridae_NOSC          DPGQMTLVFG CRQSKMDDIY KHETAQAKED GALTEVYTAL SREPGVPKSY
Branchiostoma_belcheri_NOSA          SNGDMTLVFG CRQSRVDHIY KEETAQARRD GALTDIYTAL SREPGTNKTY
Branchiostoma_belcheri_NOSB          DHGDMMLVFG CRQSETDHIY REETAQAQTD GALTDIYTAY SREPGRPKTY
Branchiostoma_belcheri_NOSC          DPGQMTLVFG CRQSKMDDIY KHETAQAKED GALTEVYTAL SREPGVPK--
Branchiostoma_lanceolatum_NOSA       TTGDMTLVFG CRQSRVDHIY KEETAQARRD GALTDLYIAL SREPGTTKTY
Branchiostoma_lanceolatum_NOSB       NHGDMTLVFG CRQSQIDHIY KDETAQAKTD GALTDIYTAY SREPDTPKTY
Branchiostoma_lanceolatum_NOSC       DPGQMTLVFG CRQSKMDDIY KHETAQAKED GALTEVYTAL SREPGVPKSY
Asymmetron_lucayanum_NOSA            TNGDMTLVFG CRQSKLDHIY KEEVAQAERD GALSDIHTAL SREPGTPKMY
Asymmetron_lucayanum_NOSB            KIGDMTLVFG CRQSKMDHIY QEETARAKKD GALTDIYTAY SREPGTPKTY
Asymmetron_lucayanum_NOSC            ---------- ---------- ---------- -VLXEVYTAL SREPGVPKTY
Ciona_intestinalis_NOS               AHCTMALLFG CRNPNHDHLY KQEVAAAMKE GAISHAYTAF SREKGKPKRY
Ciona_savignyi_NOS                   DR-TMALLFG CRNPQQDHLY KDEVETAMKQ GAISHAYTAF SREEGKPKQY
Strongylocentrotus_purpuratus_NOSa   EGGDMTLVYG CRNAKED-LY KGETSVAKDE GALTAVLTGY SREPGKPRKY
```

```
                                                951
Homo_sapiens_nNOS                       VQDILQEQLA ESVYRALEQG GHIYVCGDVT MAADVLKAIQ RIMTQQGKLS
Homo_sapiens_iNOS                       VQDILRQQLA SEVLRVLKEP GHLYVCGDVR MARDVAHTLK QLVAAKLKLN
Homo_sapiens_eNOS                       VQDILRTELA AEVHRVLLER GHMFVCGDVT MATNVLQTVQ RILATEGDME
Branchiostoma_floridae_NOSA             VQDIIRQQMP RKVLDLVRDG GHVYVCGDVT MATDVCETIQ RILVEHGGMS
Branchiostoma_floridae_NOSB             VQHVLYNQLS EKVKELLENS GHIYVCGDVA MADDVCTTVQ KILEEKSGMS
Branchiostoma_floridae_NOSC             VQNVILDLIP EKVCDLLNHN GHFYVCGDVS MAADVCNTLE KVMEKQQGMA
Branchiostoma_belcheri_NOSA             VQDILRERIP RKVLDLVREG GHVYVCGDVT MATDVCETVQ RILVKHGGMS
Branchiostoma_belcheri_NOSB             VQHILHNQLS KRVQELLDSD GHIYVCGDVA MADDVCTTVQ KILEENSAMT
Branchiostoma_belcheri_NOSC             --NVILDQIP EKVCDLLKQN GHFYVCGDVS MAADVCNTLE KVLEKQQGLT
Branchiostoma_lanceolatum_NOSA          VQDIIRQQIP KKVLDLVKDG GHIYVCGDVT MATDVGETVQ RILVEHGGMS
Branchiostoma_lanceolatum_NOSB          VQHVLYNQLS ERVKELLKNN GHIYVCGDVA MADDVCTTVQ KILEEKSAMT
Branchiostoma_lanceolatum_NOSC          VQNVILDLIP EKVCDLLKHN GHFYVCGDVS MAADVCNTLE KAMEKQQGMT
Asymmetron_lucayanum_NOSA               VQDILRQQIS RKVRDLVRDG GHIYVCGDVT MATDVCTAVQ KILEEHGGMS
Asymmetron_lucayanum_NOSB               VQHVLHNKLS KRVQELLENN GHIYVCGDVA MADDVCTTVQ KILEETSGMK
Asymmetron_lucayanum_NOSC               VQNVILDQIP QKVCDLLKHN GHFYVCGDVS MAADVCNTLE TVMEKEGGMT
Ciona_intestinalis_NOS                  VQNVIMSEIP DFVYDIVEKK GHFYVCGDVT MATEVNKTLL KILCIKGEMT
Ciona_savignyi_NOS                      VQDVIMNDIS EFVFDLITRN GHFYVCGDVT MAAEVHRTLK DVLCINGGMT
Strongylocentrotus_purpuratus_NOSa      VQDILKDH-P WLVYNVLRRQ GHIYVSGDVN MVREVSNTIE GIIAQFGRVG

                                       1001
Homo_sapiens_nNOS                       AEDAGVFISR MRDDNRYHED IFGVTLRTYE VTNRLRSIEE SKKDTDE
Homo_sapiens_iNOS                       EEQVEDYFFQ LKSQKRYHED IFGAVFPYEA KKDRVA-VQP SSLEMSA
Homo_sapiens_eNOS                       LDEAGDVIGV LRDQQRYHED IFGLTLRTQE VTSRIRTFDP PGSDTNS
Branchiostoma_floridae_NOSA             RARAEDFINT MKDNNRYHED IFGVTLKTHE VENEARKLVT SDDEDTS
Branchiostoma_floridae_NOSB             STESEAFIRN LKDSNRYHED IFGVTLNTKA VREAARSVEP ADSIANG
Branchiostoma_floridae_NOSC             PNRAKDFVDK LKDCNRYHED IFGVTLRTQE VTDRVRSVRE VGENTLI
Branchiostoma_belcheri_NOSA             AARAEDFINN MKDNNRYHED IFGVTLKTHE VEDAARKFTS VNYHFSN
Branchiostoma_belcheri_NOSB             STESEAFIRS LKDSNRYHED IFGVTLNTKA VREAARNTEP SESTANA
Branchiostoma_belcheri_NOSC             PTKAKDFVDK MKDCNRYHED IFGVTLRTQE VTDRVRSLSP SSSLSST
Branchiostoma_lanceolatum_NOSA          VARAEDFINN MKDNNRYHED IFGVTLKTHE VEDAARKISL SEENTSL
Branchiostoma_lanceolatum_NOSB          STESEALIRM LKDSNRYHED IFGVTLNTKE VREAARS--- ---VESA
Branchiostoma_lanceolatum_NOSC          PNKAKDFVDK LKDCNRYHED IFGVTLRTQE VTDRVRSVRE SSSLSST
Asymmetron_lucayanum_NOSA               AARAEDFVNN MKDNNRYHED IFGVTLKTHE VQDAARKLMV SDGDSTR
Asymmetron_lucayanum_NOSB               ADESEAFIRN LKDSNRYHED IFGVTLNTTE VREAARNVEN GESLDNG
Asymmetron_lucayanum_NOSC               AKNAKDFVDK LKDCNRYHED IFGVTLRTQE VTDRVRSVRE SSSLSST
Ciona_intestinalis_NOS                  KEKAQEFINS MKTEERYHED IFGVTLRTYE VNRSVRLKEK SESLSHK
Ciona_savignyi_NOS                      PEQVEEYITA MKSEDRYHED IFGVTLRTYE VNRSL----- -------
Strongylocentrotus_purpuratus_NOSa      AAEAKDFVRR IKDEDHYHEE MYGLTLHVSE VMEKIRAVRI EEKAVII
```

Supplementary Table S6
List of primers used for *Branchiostoma lanceolatum Nos* cloning and real time PCR experiments.

Primer name	Primer sequence		PCR product length
**Cloning**			
Nos-A	FORWARD	CTGAAGTACGCCTACGATG	1076 bp
	REVERSE	GATTTGACCAAACCAGCTCC	
Nos-B	FORWARD	CGGTACAATCCAGAGAAACG	830 bp
	REVERSE	GTTCCAGTTCCAGGGGTACG	
Nos-C	FORWARD	TCGGCCGAACGTAATTGCCG	787 bp
	REVERSE	CAGCCAGTTCTTCATGCGGGC	
**qPCR**			
Nos-A	FORWARD	AGTACAGTCATCTCCAGAAC	F-R1 = 221 bp<br>F-R2 =258 bp
	REVERSE 1	TCTTGCAGCGCTTCTATCTG	
	REVERSE 2	CGTCCATGATTTGACCAAAC	
Nos-B	REVERSE	AGTTTACTCCCGGCGATCA	191 bp
	FORWARD	AGAACATGGCGGCAAACGC	
Nos-C	REVERSE	CAGGATTCTGCGCGTTTGC	197 bp
	FORWARD	GGAGCTAGCCTCGCTCATG	
L-32	REVERSE	GGCTTCAAGAAATTCCTCGTC	117 bp
	FORWARD	AATGAGTTTCCTCTTGCGTGA	
